# “*Ca*. Nitrosocosmicus” members are the dominant archaea associated with plant rhizospheres

**DOI:** 10.1128/msphere.00821-24

**Published:** 2024-11-12

**Authors:** Ui-Ju Lee, Joo-Han Gwak, Seungyeon Choi, Man-Young Jung, Tae Kwon Lee, Hojin Ryu, Samuel Imisi Awala, Wolfgang Wanek, Michael Wagner, Zhe-Xue Quan, Sung-Keun Rhee

**Affiliations:** 1 Department of Biological Sciences and Biotechnology, Chungbuk National University, Cheongju, Republic of Korea; 2 Department of Science Education, Jeju National University, Jeju, Republic of Korea; 3 Department of Environmental Engineering, Yonsei University, Wonju, Republic of Korea; 4 Division of Terrestrial Ecosystem Research, Center of Microbiology and Environmental Systems Science, University of Vienna, Vienna, Austria; 5 Department of Microbiology and Ecosystem Science, Centre for Microbiology and Environmental Systems Science, University of Vienna, Vienna, Austria; 6 The Comammox Research Platform, University of Vienna, Vienna, Austria; 7 Department of Chemistry and Bioscience, Center for Microbial Communities, Aalborg University, Aalborg, Denmark; 8 School of Life Sciences, Fudan University, Shanghai, China; University of California at Davis, Davis, California, USA

**Keywords:** rhizosphere, ammonia-oxidizing archaea, catalase, nitrification

## Abstract

Archaea catalyzing the first step of nitrification in the rhizosphere possibly have an influence on plant growth and development. In this study, we found a distinct archaeal community, dominated by ammonia-oxidizing archaea (AOA), associated with the root system of pepper (*Capsicum anuum* L.) and ginseng plants (*Panax ginseng* C.A. Mey.) compared to bulk soil not penetrated by roots. While the abundance of total AOA decreased in the rhizosphere soils, AOA related to “*Candidatus* Nitrosocosmicus,” which harbor gene encoding manganese catalase (MnKat) in contrast to most other AOA, dominated the AOA community in the rhizosphere soils. For both plant species, the ratio of copy numbers of the AOA MnKat gene to the *amoA* gene (encoding the ammonia monooxygenase subunit A) was significantly higher in the rhizospheres than in bulk soils. In contrast to MnKat-negative strains from other AOA clades, the catalase activity of a representative isolate of “*Ca.* Nitrosocosmicus” was demonstrated. Members of this clade were enriched in H_2_O_2_-amended bulk soils, and constitutive expression of their MnKat gene was observed in both bulk and rhizosphere soils. Due to their abundance, *“Ca.* Nitrosocosmicus*”* members can be considered important players mediating the nitrification process in rhizospheres. The dominance of this MnKat-containing AOA in rhizospheres of agriculturally important plants hints at a previously overlooked AOA-plant interaction.

**IMPORTANCE:**

Ammonia-oxidizing archaea (AOA) are widespread in terrestrial environments and outnumber other ammonia oxidizers in the rhizosphere, possibly exerting an influence on plant growth and development. However, little is known about the selection forces that shape their composition, functions, survival, and proliferation strategies in the rhizosphere. Here, we observed a distinct AOA community on root systems of two different plant species compared to bulk soil. Our results show that the *“Ca.* Nitrosocosmicus*”* clade, which possesses functional MnKat genes unlike most other AOA, dominated the rhizosphere soils. Moreover, members of this clade were enriched in H2O2-amended bulk soil, which mimics the ROS stress in root systems. While research on AOA-plant interactions in the rhizosphere is still in its infancy, these findings suggest that *“Ca.* Nitrosocosmicus*”* may be an important clade of AOA with potential AOA-plant interaction.

## INTRODUCTION

Rhizosphere microbiome has been proposed to confer specific functions to its host plant, by modulating plant nutrient uptake, stress resistance, growth, and health (1–3). Soil types and characteristics are primarily shown to determine the background (bulk soil) microbiome, from which rhizosphere microbiomes are selected (4–9). Due to rhizodeposition, rhizospheres exhibit higher microbial abundances and distinct microbial communities compared to bulk soil (10–13).

Plant root exudates influence rhizosphere microbial community development by stimulating or inhibiting specific types of microorganisms (9, 14–16). Depending on the mode of photosynthesis (17) as well as the physiological and developmental status of the plant (18–20), roots release different types of exudates into the rhizosphere. Also, the phylogeny and genotype of a plant contribute to the development of distinct microbial communities in the plant rhizosphere by influencing the composition and activity of root exudates (13, 21–24). Conversely, it has been demonstrated that the rhizosphere microbiome affects root exudation inversely (2). Furthermore, it has been postulated that plants actively recruit soil microorganisms by releasing specific compounds into their rhizosphere that selectively stimulate specific microorganisms that are beneficial to plant growth and health (25–27). Signal molecules and antimicrobial compounds found in root exudates, such as phytoanticipins, phytoalexins, and sorgoleone, can be critical factors for shaping rhizosphere microbial communities (28–31). While we have gained a better understanding of the biology of root development as well as the structure and function of microbial communities in the rhizosphere, the interactions between rhizosphere microbiomes and plant roots *via* exudate secretion are not well understood (32).

Many studies on the rhizosphere microbiome have been conducted over the years. However, only a few of them have focused on the archaeal microbiome of roots (33–37). Although archaea are widespread in terrestrial environments (38–41), little is known about the selection forces that shape their composition as well as their functions, survival, and proliferation strategies in the rhizosphere (42). *Nitrososphaerota* (formerly known as Thaumarchaeota) are the predominant archaea found in soil (40, 43). Members of *Nitrososphaerota* belonging to groups I.1a, I.1a-associated, and I.1b (44) are ammonia-oxidizing archaea (AOA) involved in autotrophic ammonia oxidation, a key step in the nitrification process (43). Nitrification changes the availability of nitrogen species to plants and thus affects nitrogen fertilizer efficiency and enhances nitrogen mobility in the environment, resulting in fertilizer loss and eutrophication of water bodies. In addition, the nitrification intermediate, nitric oxide, functions as a signaling molecule in plants (45), and ammonia-oxidizing microorganisms (AOM) produce and emit nitrous oxide from agricultural soil (46).

Here, we analyzed archaeal communities associated with the rhizosphere of pepper and ginseng plants. The majority of the archaea identified in bulk and rhizosphere soils were AOA related, and they were found to outnumber ammonia-oxidizing bacteria (AOB) in both bulk and rhizosphere soils. Furthermore, AOA communities differed between bulk and rhizosphere soils, with the latter dominated by AOA closely related to “*Candidatus* Nitrosocosmicus,” a known manganese catalase (MnKat)-containing AOA. Based on these findings, we propose that *“Candidatus* Nitrosocosmicus*”* may be an important AOM associated with the rhizosphere.

## RESULTS

### 16S rRNA gene amplicon-based archaeal community analysis in pepper and ginseng rhizosphere soils

The prokaryotic communities in bulk and rhizosphere soils of pepper plants were examined using 16S rRNA gene amplicon sequencing during the plant vegetative (60-day-old) and reproductive (90-day-old) growth phases. The compositions and diversities of prokaryotic communities in pepper plant rhizosphere soils differed from those in bulk soils regardless of growth phase (see more details in Supplementary Results and Discussion) (Fig. S1). Archaea were less abundant in rhizosphere soils (0.66 ± 0.23%; *n* = 10) compared to bulk soils (3.46 ± 0.64%; *n* = 15) in both plant growth phases (Table S1). Members of the phylum *Nitrososphaerota*, which were abundant in both the rhizosphere and bulk soils, accounted for a significant proportion of total archaeal 16S rRNA gene reads (94.2%; *n* = 25). They were also the 11th most abundant prokaryotic phyla detected (Fig. S1A; Table S1), with the majority of their 16S rRNA gene amplicon sequencing variants (ASVs) belonging to three AOA groups (I.1a, I.1a-associated, and I.1b). When compared to other AOM, such as *Nitrosomonadaceae* (the family that includes AOB) and *Nitrospiraceae* [the family that includes both complete ammonia oxidizers (Comammox) and nitrite oxidizers], ASVs belonging to AOA were the most abundant in both bulk and rhizosphere soils of pepper plants (Fig. S2A).

**Fig 1:**
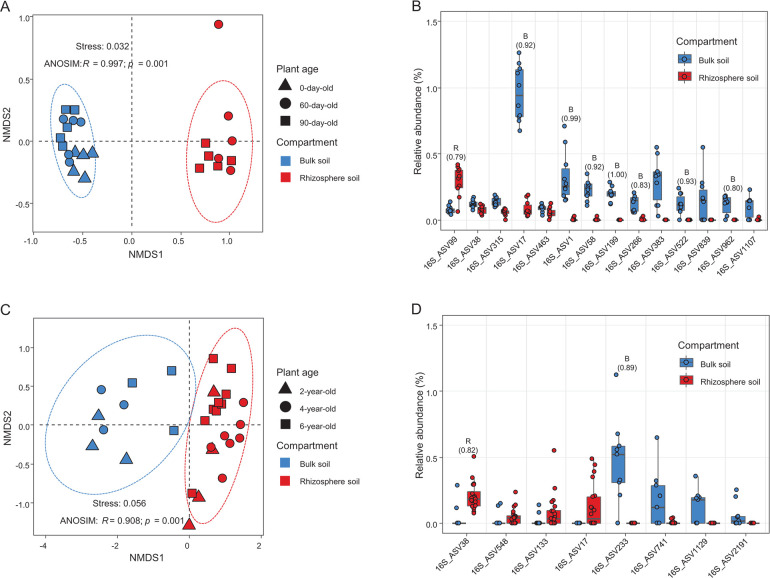
Distinct AOA communities in bulk and rhizosphere soils of pepper and ginseng plants using 16S rRNA gene primer. (A and C) NMDS plot using Bray–Curtis dissimilarity metrics of AOA communities in bulk and rhizosphere soils, based on AOA 16S rRNA gene profiles of pepper and ginseng plants. In pepper plants, a total of 25 samples were collected: bulk soil samples (*n* = 5) at day 0, and bulk (*n* = 5) and rhizosphere soil samples (*n* = 5) at both 60 and 90 days (A). For ginseng plants, a total of 29 samples were collected: bulk soil samples (*n* = 3) and rhizosphere soil samples (*n* = 4, 7, 9) from 2-, 4-, and 6-year-old plants, respectively (C). (B and D) Relative abundances (% of the total 16S rRNA gene reads) of AOA 16S rRNA gene ASVs, including only those with a mean relative abundance of ≥0.01% across all samples, are shown for pepper plants (B) and ginseng plants (D). Results of indicator species analysis are shown above each ASV’s bar with the IndVal value in parenthesis. Indicator values (IndVal > 0.75, q < 0.01) are displayed for rhizosphere (R) or bulk soil (B).

**Fig 2:**
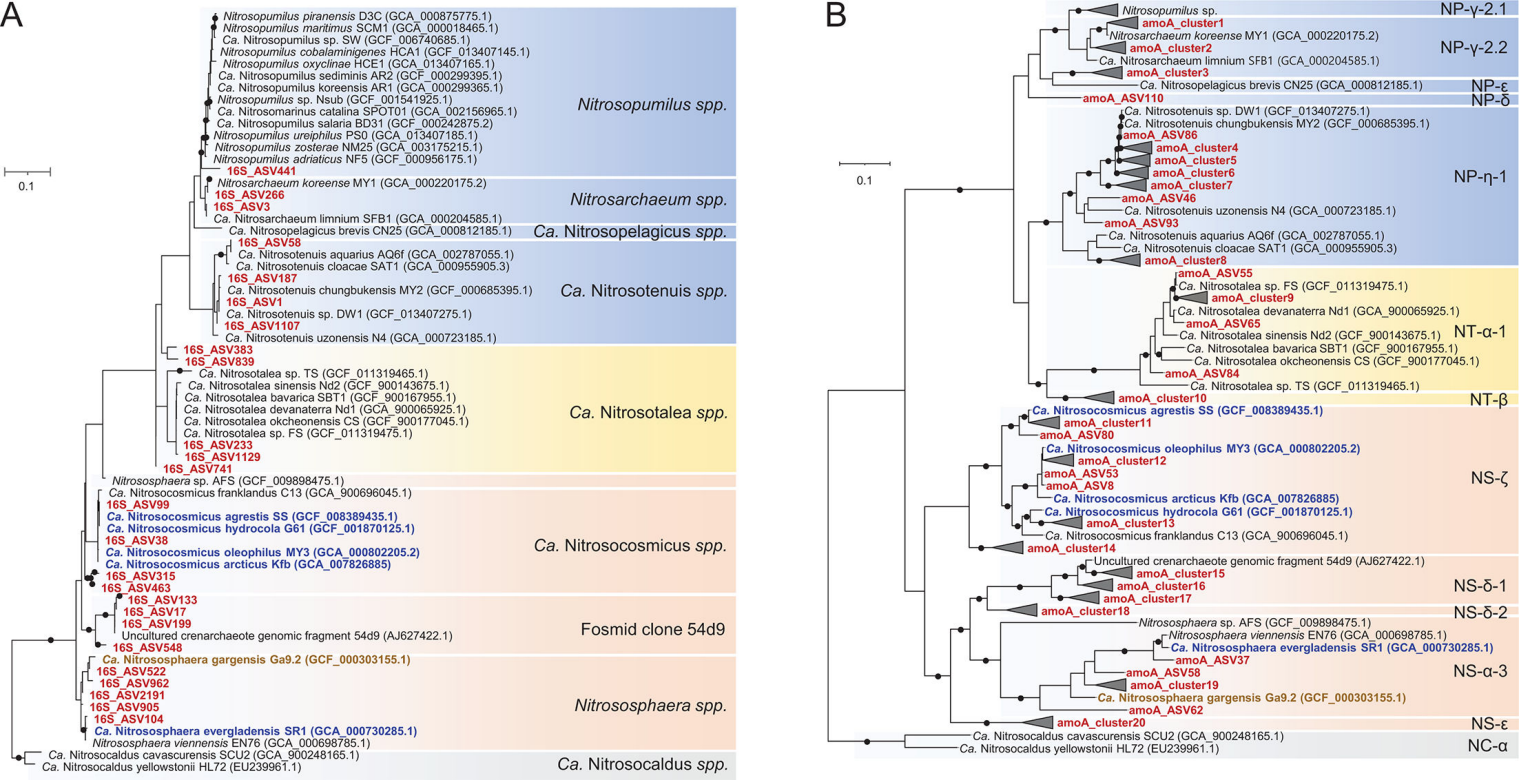
Maximum likelihood phylogenetic trees of AOA 16S rRNA and amoA genes. (A) Phylogenetic tree of AOA 16S rRNA gene sequences reconstructed using IQ-TREE. The GTR + F + I + G4 model was determined using ModelFinder Plus (47) within IQ-TREE (48). (B) Phylogenetic tree of AOA amoA genes reconstructed using IQ-TREE. The TIM2+F + R5 model was determined using ModelFinder Plus (47) within IQ-TREE (48). ASVs with a relative abundance of more than 0.01% at 16S rRNA and 0.1% at AOA amoA were used for the analysis. ASVs with more than 96% sequence similarity on nucleotide level were grouped into single cluster, and cluster information is displayed in Data Set S1. Branch supports for 1,000 replicates were obtained using the ultrafast bootstrap and SH-aLRT tests. Branch supports ≥95% are indicated by black circles. MnKat gene-containing AOA are depicted in bold and blue letters. A truncated MnKat gene-containing AOA is shown in bold and brown letters. Each AOA group is indicated by different colored backgrounds (group I.1a, blue; group I.1a-associated, yellow; group I.1b, red; and thermophilic AOA, gray).

Interestingly, distinct AOA communities were observed on the non-metric multidimensional scaling (NMDS) plot between bulk and rhizosphere soils (Fig. 1A), with a low-stress value (Stress = 0.032), and ANOSIM analysis supported this difference (*R* = 0.997, *P* < 0.001). The difference between plant growth phases had no significant effect on the AOA communities in the rhizosphere soils (*R* = 0.033, *P >* 0.05). To identify ASVs that contributed to the discrimination of the AOA communities between bulk and rhizosphere soils, an indicator species analysis was performed at the ASV level. Among the AOA 16S rRNA gene ASVs, only 16S_ASV99 was significantly more abundant in the rhizosphere soils (IndVal: 0.79, q < 0.01) (Fig. 1B). This ASV is closely related to members of the clade *“Ca.* Nitrosocosmicus*”* in group I.1b (Fig. 2A), with >99.5% 16S rRNA sequence similarity. A comparison of the relative abundances of the AOA 16S rRNA gene ASVs showed that 16S_ASV99 was the single most abundant AOA ASV in the rhizosphere soils (an average of 44.4% of the total AOA 16S rRNA gene reads) (Fig. 1B). On the other hand, two AOA 16S rRNA gene ASVs were dominant in the bulk soils (16S_ASV17 and 16S_ASV1, accounting for 29.4% and 8.8%, respectively, of the total AOA 16S rRNA gene reads) (Fig. 1B), and they were closely related to fosmid clone 54d9 of group I.1b and the clade *“Ca.* Nitrosotenuis*”* of group I.1a, respectively (Fig. 2A).

For comparison, prokaryotic communities in bulk and rhizosphere soils of perennial ginseng plants (2-, 4-, and 6-year-old), collected from a different geographical region than the pepper plants, were analyzed (Table S2). As observed for pepper plants (Fig. S1A; Table S1), AOA were the most dominant archaea in both bulk and rhizosphere soils (Table S2). Rhizosphere AOA communities of the ginseng plants clustered based on the AOA 16S rRNA gene profiles (Fig. 1C) but were distinct from those of the bulk soils (Stress: 0.056; ANOSIM, *R* = 0.908, *P* < 0.001). The plant age had no significant effect on the AOA communities in the rhizosphere soils (ANOSIM, *R* = 0.158, *P* < 0.05). Notably, 16S_ASV38 related to *“Ca.* Nitrosocosmicus*”* was the most abundant ASV (accounting for 48.2% of the total AOA 16S rRNA gene reads) in the rhizosphere of the ginseng plants and strongly associated with rhizosphere soils than bulk soils (IndVal: 0.82, q < 0.01) (Fig. 1D and 2A) as observed in the pepper plants. In contrast to the pepper plants, two AOA ASVs were dominant in the bulk soils (16S_ASV233 and 16S_ASV741, accounting for 37.7% and 12.9%, respectively, of the total AOA 16S rRNA gene reads (Fig. 1D), and were closely related to the clade *“Ca.* Nitrosotalea*”* of group I.1a-associated (Fig. 2A).

### AOA *amoA* gene amplicon-based AOA community analysis

**Fig 3:**
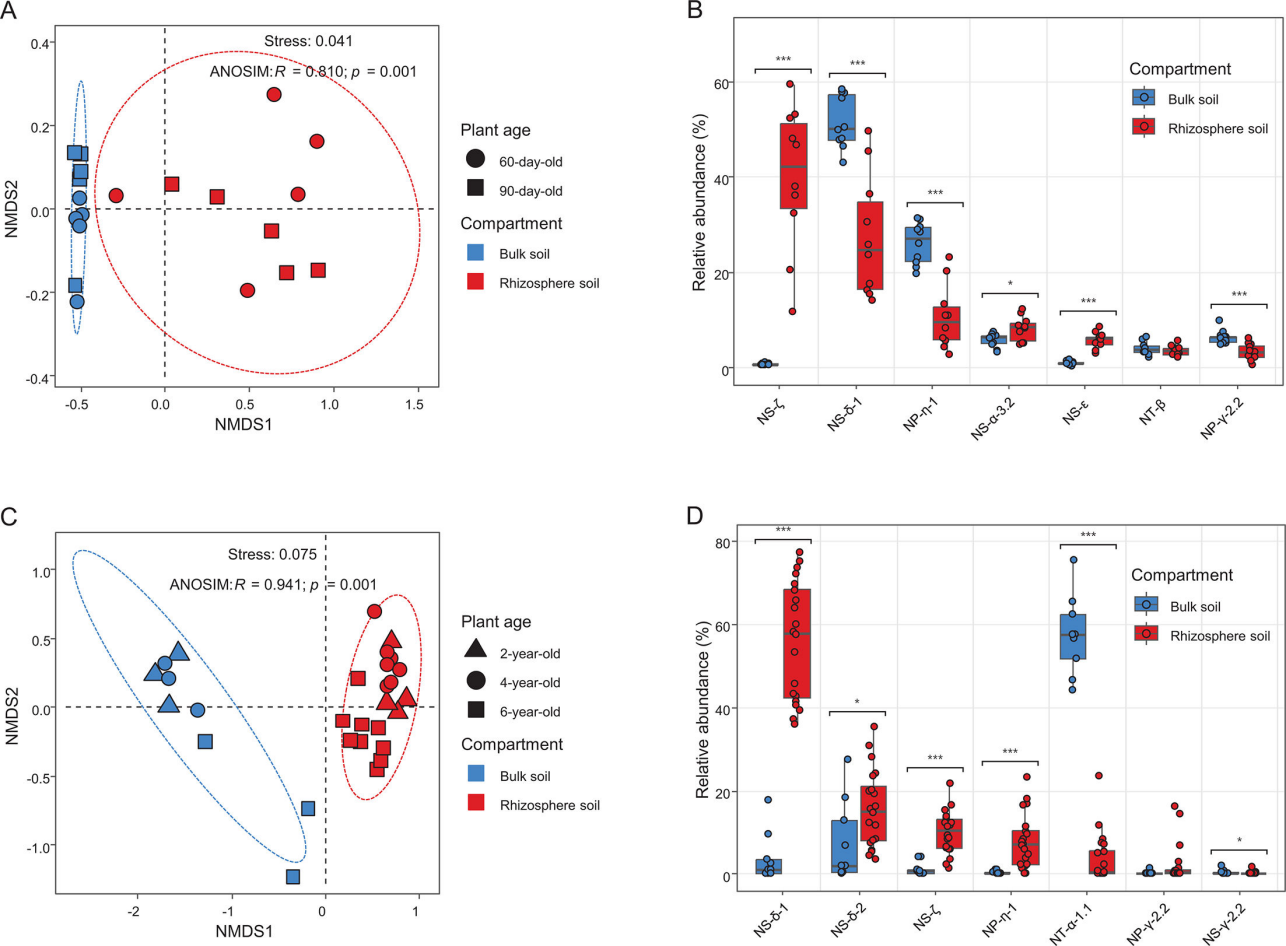
Distinct AOA communities in bulk and rhizosphere soils of pepper and ginseng plants using AOA *amoA* gene primer. (A and C) NMDS plot using Bray–Curtis dissimilarity metrics of AOA communities in bulk and rhizosphere soils, based on AOA *amoA* gene profiles of pepper and ginseng plants. In pepper plants, a total of 25 samples were collected: bulk soil samples (*n* = 5) at day 0, and bulk (*n* = 5) and rhizosphere soil samples (*n* = 5) at both 60 and 90 days (A). For ginseng plants, a total of 29 samples were collected: bulk soil samples (*n* = 3) and rhizosphere soil samples (*n* = 4, 7, 9) from 2-, 4-, and 6-year-old plants, respectively (C). (B and D) Relative abundance (% of each AOA clade relative abundances as the sum of AOA *amoA* ASV abundances) of AOA clade, including only those with a mean relative abundance of ≥0.1% across all samples, are shown for pepper plants (B) and ginseng plants (D). Statistical significance was determined using Student’s *t*-tests (**P < 0.005; ***P < 0.0005).

The AOA *amoA* gene amplicon sequencing analysis supported the presence of distinct AOA communities in the rhizosphere vs bulk soils of pepper plants (Stress: 0.041; ANOSIM: *R* = 0.810, *P* < 0.001), while no significant difference was observed based on plant age (ANOSIM: *R* = −0.01, *P* > 0.05) (Fig. 3A). The number of *amoA* gene reads was summed for each AOA clade and compared between bulk and rhizosphere soils (Fig. 3B). Two *“Ca.* Nitrosocosmicus*”*-related *amoA* ASV clusters (amoA_cluster11, and −13) of clade NS-ζ (Zeta) (49) (Fig. 2B; Data set S1) had the highest relative abundance (average 39.7%; *n* = 10) (Fig. 3B) and strongly contributed to the clustering of the AOA communities in rhizosphere soils (Fig. 3A). On the other hand, the two most abundant bulk soil-associated AOA clade were NS-δ (Delta)−1 and NP-η (Eta)−1, respectively (Fig. 3B; Data set S1). These *amoA* clades correspond to the AOA clades fosmid clone 54d9 and *“Ca.* Nitrosotenuis,” respectively (Fig. 2B). These *amoA*-based results are highly consistent with the AOA 16S rRNA gene profiles (Fig. 1A and B).

In ginseng plants, the AOA *amoA* gene amplicon sequencing analysis also confirmed the clear segregation of the AOA communities between bulk and rhizosphere soils (Stress: 0.075; ANOSIM, *R* = 0.941, *P* < 0.001), as observed in pepper plants (Fig. 3A). However, plant age showed no significant effect on AOA communities (ANOSIM, *R* = 0.233, *P* < 0.01) (Fig. 3C). The relative abundance of the clade NS-ζ (amoA_ASV8, −34, and amoA_cluster12) significantly increased in the rhizosphere soils, while that of the clade NT-α−1 (i.e., I.1a-associated; amoA_cluster9) decreased (Fig. 3D; Data set S1), which was consistent with the results of the AOA 16S rRNA gene analysis (Fig. 1D). However, the notable increase in the relative abundance of the clades NS-δ−1 (amoA_cluster15, −16, and −17) and NP-η−1 (amoA_cluster5, −7 and −8) in the ginseng rhizosphere soils (Fig. 3D; Data set S1) was unexpected considering the AOA 16S rRNA gene analysis results (Fig. 1D).

### Abundance of AOA MnKat gene in rhizosphere soils

**Fig 4:**
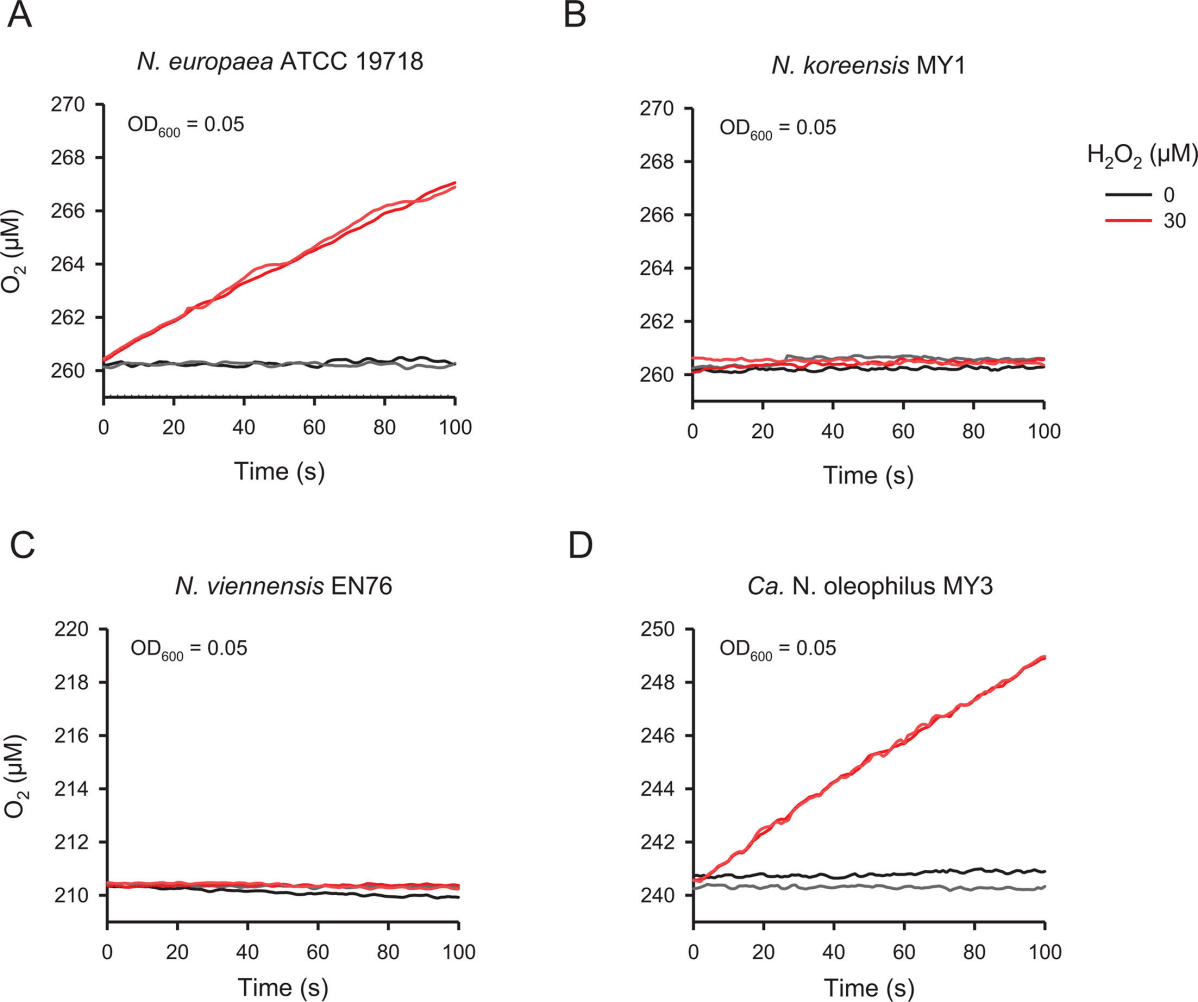
Catalase activity of AOB and AOA strains. An assessment of oxygen production in the presence of 30 µM H_2_O_2_ in an AOB strain (A) and three AOA strains (B–D). The results of duplicate experiments are shown.

Members of the genus “*Ca.* Nitrosocosmicus” possess genomic, morphological, and physiological properties distinct from other AOA (50–53). They possess genes that encode a putative MnKat (50–53) (Fig. S3). Despite the presence of MnKat genes in their genomes, catalase activity in these AOA has not yet been directly demonstrated experimentally. Nonetheless, the ability of “*Ca*. Nitrosocosmicus oleophilus” to grow in the absence of H_2_O_2_ scavengers, unlike other AOA, has already highlighted the presence of an active MnKat (50, 54). To test for catalase activity, we performed whole-cell assays with different AOMs. As expected, the heme-catalase-containing AOB strain (*Nitrosomonas europaea* ATCC 19718) (55), which was used as a positive control, generated O_2_ in the presence of H_2_O_2_ (Fig. 4A). Similarly, O_2_ generation in the presence of H_2_O_2_ was detected for “*Ca*. Nitrosocosmicus oleophilus” MY3 (Fig. 4B), consistent with the proposed activity of its MnKat. By contrast, O_2_ generation was not observed in the tested catalase gene-negative AOA, *Nitrosarchaeum koreense* MY1 (clade NP-γ (Gamma)−2.2; group I.1a), and *Nitrososphaera viennensis* EN76 (clade NS-α (Alpha)−3; group I.1b) (Fig. 4C and D). Based on these observations, we hypothesized that catalase activity can be one of the important factors associated with the dominance and survival of the “*Ca.* Nitrosocosmicus”-related AOA in the rhizosphere AOA community of pepper and ginseng plants. Coincidently, catalase-containing bacterial 16S rRNA gene ASVs were abundant in the rhizosphere soils of the pepper plants compared to bulk soils (Fig. S4; Data set S2 and S3). Specifically, 68.5% of the top 73 rhizosphere soil-associated ASVs (indicated by a positive value in log_2_fold) are known to have catalase genes or activity. By contrast, only 22.4% of the top 58 bulk soil-associated ASVs (indicated by a negative value in log_2_fold) have a catalase gene or activity (Fig. S4; Data set S2 and S3).

**Fig 5:**
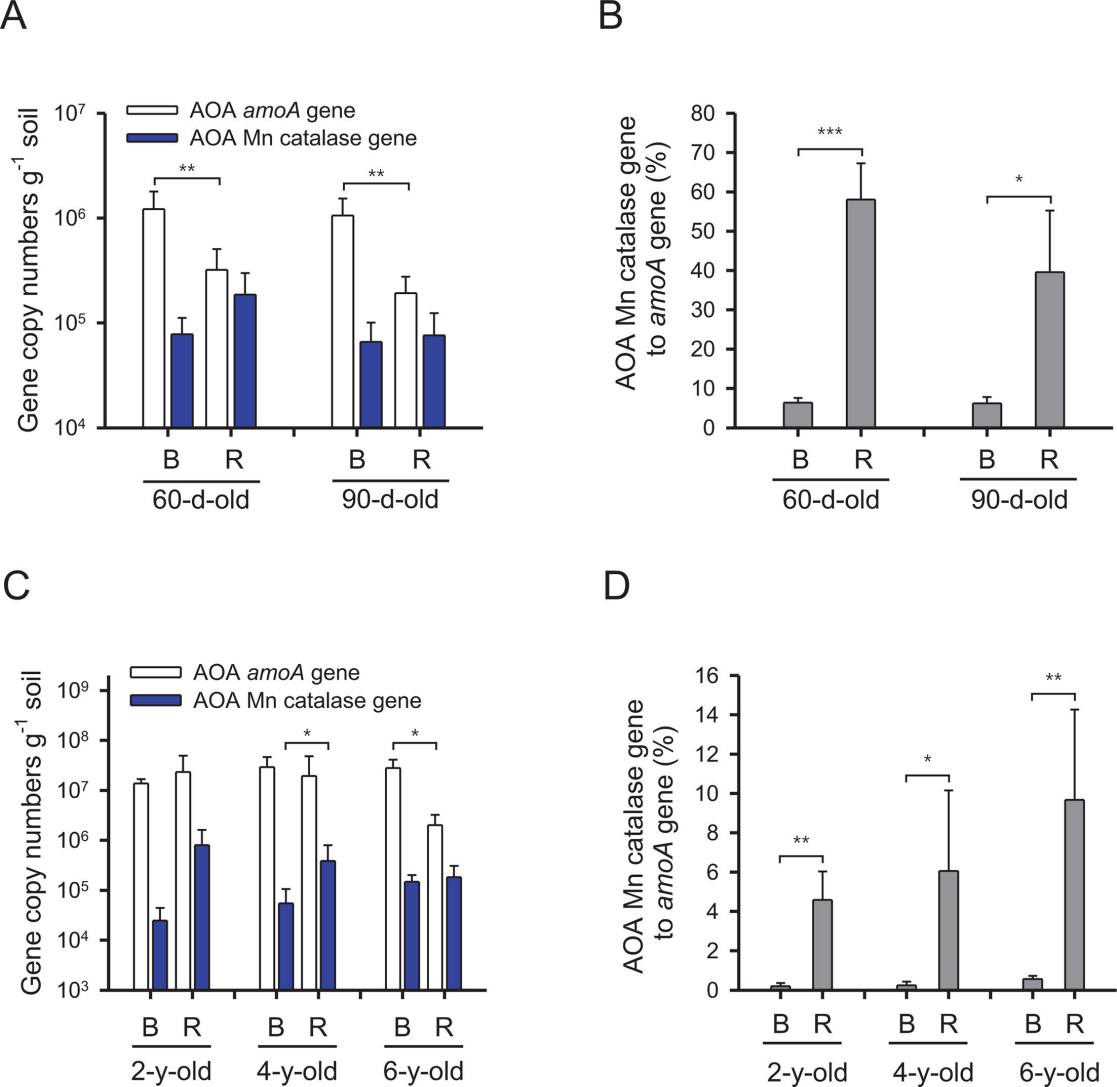
Abundance of AOA *amoA* and MnKat genes. (A) Copy numbers of AOA *amoA* and MnKat genes in each soil compartment at two different growth phases of the pepper plants (60- and 90-day-old). Error bars represent the standard deviations from bulk soil samples (B, *n* = 5) and rhizosphere soil samples (R, *n* = 5). (B) The copy number ratios (%) of the AOA MnKat gene to the *amoA* gene calculated from (A) are shown. (C) The copy numbers of AOA *amoA* and MnKat genes in each soil compartment at three different ages (2-, 4-, and 6-year-old). Error bars represent the standard deviations of biological replicates, with bulk soil samples (B, *n* = 3 for each age) and rhizosphere soil samples (R, *n* = 4, 7, and 9 for 2-, 4-, and 6-year-old plants, respectively). (D) The copy number ratios (%) of the AOA MnKat gene to the *amoA* gene calculated from (C) are shown. Statistical significance was determined using Student’s *t*-tests (*P < 0.05; **P < 0.005; ***P < 0.0005).

Thus, using qPCR, we quantified the relative abundance of MnKat-containing AOA in the rhizosphere soils of pepper and ginseng plants by comparing the copy numbers of the AOA MnKat genes relative to the *amoA* genes. Based on the newly designed PCR primer pair targeting AOA MnKat genes (see details in Materials and Methods), we found that all amplified MnKat gene sequences belonged to AOA, and more importantly, sequences associated with *“Ca*. Nitrosocosmicus”-related AOA were predominant in the rhizosphere and bulk soils (Table S3; Data set S4). In addition, AOA *amoA* gene copy numbers per gram of soil were lower in rhizosphere soils compared to bulk soils of the pepper plants (Fig. 5A). On the other hand, AOA MnKat gene copy numbers in 60- and 90-day-old rhizosphere soils were comparable to those in bulk soils (Fig. 5A). Overall, the copy number ratios of AOA MnKat genes to *amoA* genes were significantly higher in pepper plant rhizosphere soils than in bulk soils (Fig. 5B). In ginseng plants, the AOA *amoA* gene copy numbers per gram of soil were lower in rhizosphere soils compared to bulk soils only in 6-year-old plants, with similar AOA MnKat gene copy numbers in both soil types (Fig. 5C). In 4-year-old ginseng plants, AOA MnKat gene copy numbers were higher in rhizosphere soils than in bulk soils (Fig. 5C). The copy number ratios of AOA MnKat genes to *amoA* genes were consistently higher in rhizosphere soils than in bulk soils across all plant ages of ginseng plants (Fig. 5D). The results of AOA MnKat gene sequencing showed that, while sequences associated with the “*Ca.* N. oleophilus” MY3-like clade accounted for approximately 66%, and sequences associated with “*Ca.* N. everglandensis” SR1-like made up most of the remaining portion in bulk soil, in rhizosphere soils, the proportion of sequences associated with the “*Ca.* N. oleophilus” MY3-like clade increased to nearly 99% (Table S3). Overall results from both plants revealed that (i) most of the amplified MnKat gene sequences are associated with “*Ca.* Nitrosocosmicus” and (ii) the copy number ratios AOA MnKat genes to *amoA* genes were higher in the rhizosphere soils than in bulk soils.

### Enrichment of MnKat-containing AOA from soil by H_2_O_2_ treatment

To investigate the selective enrichment of MnKat-containing AOA in H_2_O_2_-amended soils, bulk soil slurries from the pepper plant experiment were incubated with 1.5 mM ammonium chloride in the presence of 0–30 µM H_2_O_2_ (Fig. S5). Because H_2_O_2_ is rapidly decomposed by soil microorganisms and abiotic processes (Fig. S6), resulting in a short half-life (14.9 min at 10 µM H_2_O_2_), it was added to the soil slurries twice daily during the incubation period. Ammonia oxidation was gradually inhibited as the concentration of amended H_2_O_2_ in the soil slurry increased (Fig. S5). The copy number ratios of AOA MnKat genes to *amoA* genes increased in proportion to the concentration of H_2_O_2_ amended (Fig. S7), indicating that the presence of H_2_O_2_ in the soil selectively enriched catalase-containing AOA.

**Fig 6:**
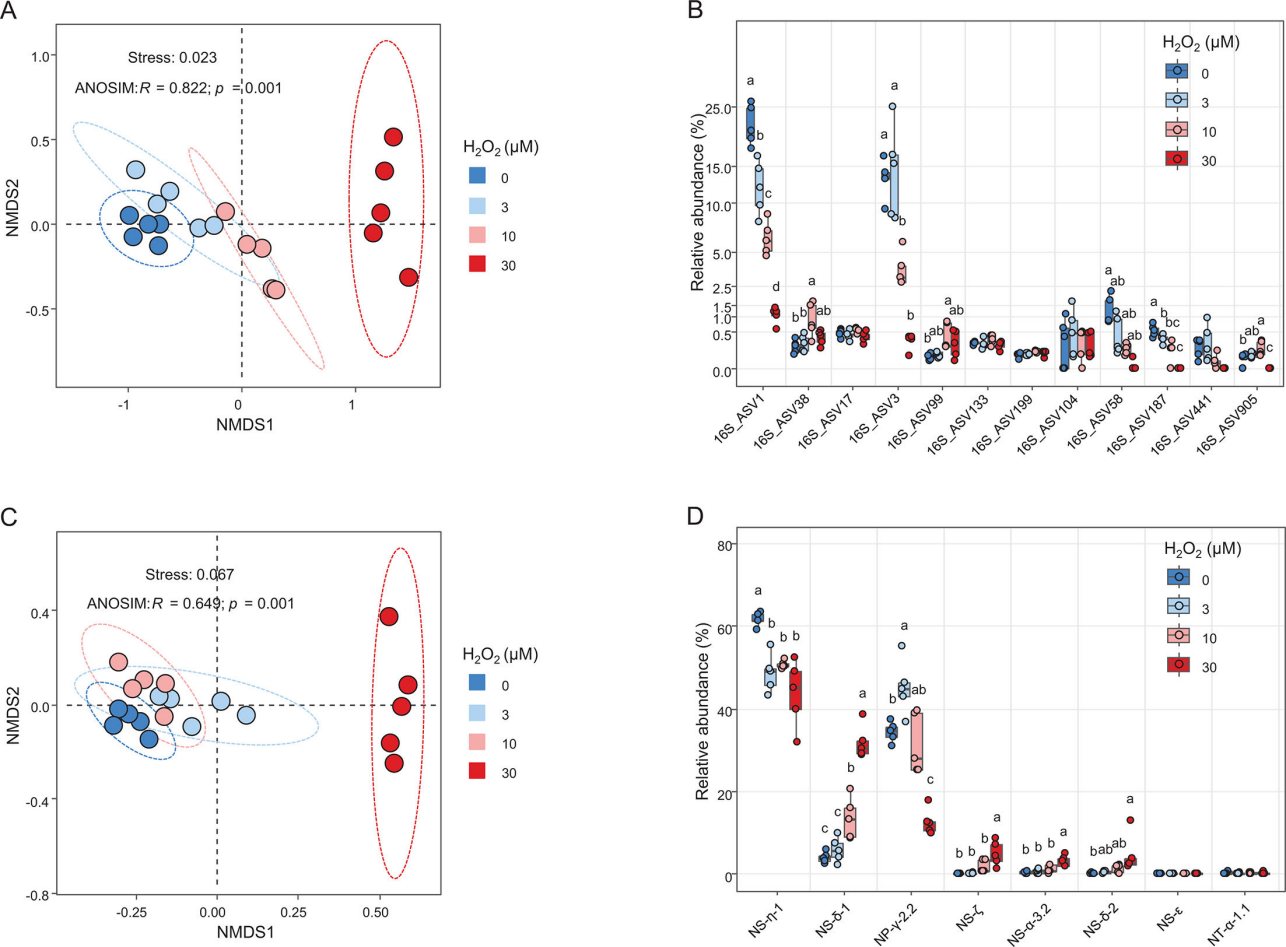
Distinct AOA communities in soil slurries amended with different H_2_O_2_ concentrations. (A) NMDS plot using Bray–Curtis dissimilarity metrics to assess AOA communities in soil slurries with different H_2_O_2_ concentrations (total *n* = 20; *n* = 5 for each H_2_O_2_ concentration), based on AOA 16S rRNA gene profiles. (B) Relative abundances (% of the total 16S rRNA gene reads) of the top 12 AOA 16S rRNA ASVs, including only those with a mean relative abundance of ≥0.1% across all samples, are shown. Significant differences in the relative abundance of each ASV between soil slurries are indicated by different letters (one-way ANOVA, Tukey’s test, *P* < 0.05). The phylogenetic affiliation of the ASVs is shown in Fig. 3A. (C) NMDS plot using Bray–Curtis dissimilarity metrics to assess AOA communities in soil slurries with different H_2_O_2_ concentrations (total *n* = 20; *n* = 5 for each H_2_O_2_ concentrations), based on AOA *amoA* gene profiles. (D) The sum of the percent relative abundance of *amoA* gene ASVs of each AOA clade (>0.1% of the mean of relative abundances). Significant differences in the relative abundance of each ASV between soil slurries are indicated by different letters (one-way ANOVA, Tukey’s test, *P* < 0.05).

AOA community analysis of the soil slurries amended with H_2_O_2_ was performed using amplicon sequencing of prokaryotic 16S rRNA and AOA *amoA* genes (Fig. 6). We found a clear segregation between the slurry samples with different H_2_O_2_ concentrations based on the AOA 16S rRNA gene profiles (Stress: 0.023; ANOSIM, *R* = 0.822, *P* < 0.001). A cluster of the soil slurries with 30 µM H_2_O_2_ was especially distinct from the others (Fig. 6A). The relative abundances of 16S_ASV38 and −99 belonging to the clade “*Ca*. Nitrosocosmicus” (Fig. 3A) increased proportionally to the H_2_O_2_ concentration based on the AOA 16S rRNA gene amplicon sequencing results (Fig. 6B). By contrast, the relative abundance of other AOA 16S rRNA gene ASVs (16S_ASV1, -2, and -58), belonging to the clades *Nitrosarchaeum* and “*Ca*. Nitrosotenuis” of group I.1a (Fig. 3A), significantly decreased as the concentration of H_2_O_2_ increased (Fig. 6B). The relative abundance of 16S_ASV17, affiliated with the clade fosmid clone 54d9, remained unchanged across all H_2_O_2_ concentrations (Fig. 6B).

The segregation of AOA communities in the soil slurry samples with varying H_2_O_2_ concentrations was also supported by AOA *amoA* gene amplicon sequencing data (Stress: 0.067; ANOSIM, *R* = 0.649, *P* < 0.001) (Fig. 6C). The soil slurry sample cluster with 30 µM H_2_O_2_ was especially distinct from the others (Fig. 6C); this finding was also consistent with the AOA 16S rRNA gene-based analysis (Fig. 6A). The relative abundances of *amoA* clades NS-δ−1, NS-ζ, NS-α−3.2, and NS-δ−2 increased as H_2_O_2_ concentrations increased (Fig. 6D). Both the AOA 16S rRNA and *amoA* gene profiles revealed a significant increase in the relative abundance of “*Ca.* Nitrosocosmicus”-related AOA (clade NS-ζ) (Fig. 6B and D). Surprisingly, as the H_2_O_2_ concentrations increased, so did the relative abundance of clade NS-δ−1 *amoA* genes (related to the *amoA* gene on the fosmid clone 54d9), contrary to what was observed in the AOA 16S rRNA gene profile analysis (Fig. 6B). On the other hand, the relative abundance of other AOA clades in group I.1a (*amoA* clades NP-η-1 and NP-γ-2.2) decreased with increasing H_2_O_2_ concentrations (Fig. S6D), as observed in the AOA 16S rRNA gene profile analysis (Fig. 3A; Fig. 6B). Taken together, these results support the hypothesis that resistance to H_2_O_2_ is important in the selection of MnKat-containing AOA in soil habitats with elevated H_2_O_2_ concentrations, such as the plant rhizosphere.

### Expression of AOA MnKat gene in rhizosphere soils

To demonstrate AOA MnKat gene expression in rhizosphere soils, transcripts of AOA MnKat genes were quantified using cDNA generated from mRNA extracted from pepper plant rhizosphere soils and bulk soils. In addition, a *“Ca*. Nitrosocosmicus” clade-specific housekeeping gene, *rpoB*, and AOA *amoA* gene were quantified to estimate the relative abundance of AOA MnKat gene transcripts in rhizosphere soils. The RT-qPCR data revealed that the relative abundance of AOA MnKat gene transcripts to AOA *amoA* and “*Ca*. Nitrosocosmicus” clade *rpoB* gene transcripts in rhizosphere soils was not significantly different from those in bulk soils (Fig. S8), indicating possible constitutive expression of AOA MnKat genes in soils.

## DISCUSSION

There have been extensive studies on rhizosphere microbial communities, as the rhizosphere microbiome affects the survival of plants under stress conditions such as those caused by climate change, pathogen infection, etc. (1–3). Despite their potential importance in plant growth and development, archaea are only rarely included in rhizosphere microbiome studies (33–37). AOA are especially abundant in terrestrial environments and play a key role in the soil nitrogen cycle (33, 40, 43), necessitating additional research into their interactions with plant roots (33, 40). Patterns of prokaryotic communities formed in the analyzed rhizosphere soils (see more details in Supplementary Results and Discussion) (Fig. S1; Tables S1 and S2) distinct from the bulk soils of pepper plants were consistent with previous studies on other plant species (4, 8, 9, 56). Furthermore, distinct AOA communities in rhizosphere soils of pepper and ginseng plants relative to bulk soils were revealed by 16S rRNA and *amoA* genes amplicon sequencing profiles (Fig. 1A, C, 2A and C), indicating a niche differentiation of AOA between bulk and rhizosphere soils of the plants.

We observed a decrease in the relative abundance of AOA in rhizosphere soils of pepper and ginseng plants compared to bulk soils (Fig. S1A; Tables S1 and S2), which contradicts previous findings (57, 58). Although AOA had a low relative abundance in rhizosphere soils of pepper and ginseng plants, they still outnumbered other AOM (Fig. S2). Despite extensive studies on ecology of AOM in various soils, only limited studies were conducted for ecological factors affecting niche differentiation of clades of AOM in rhizosphere soils. It was known that traits of plants and N-fertilization can affect the relative abundance of AOA to AOB in the rhizospheres of the plants compared to AOB (58–60). Further research on biotic and abiotic rhizosphere factors affecting the differential abundance of AOA and AOB is warranted. Consequently, a low abundance of AOM may decrease nitrification activity near plant roots, which is desirable to reduce N losses and increase N fertilizer use efficiency (59, 61).

AOA related to “*Ca*. Nitrosocosmicus” were notably the most abundant in the rhizosphere soils based on 16S rRNA and *amoA* gene amplicon analyses (Fig. 1B, D, 2, 3B and 3D; Data set S1). Interestingly, among genome-sequenced AOA, MnKat genes are exclusively present in members of the genus “*Ca.* Nitrosocosmicus” and of the species “*Ca.* Nitrososphaera evergladensis” (50–52, 62) (Fig. 2). Phylogenetic analysis of MnKat genes (Fig. S3) revealed that *Nitrososphaerota* MnKat genes were closely related to those found in the bacterial phylum *Terrabacteria*, which includes common soil bacteria such as *Actinomycetota* and *Bacillota* (53) (Fig. S3). In addition, these genes differed from those found in closely related archaeal phyla, *Ca.* Thermoproteota and *Euryarchaeota*, implying that horizontal gene transfer events between archaea and bacteria shaped the evolutionary history of the MnKat gene (Fig. S3).

Catalase activity was measured in “*Ca.* Nitrosocosmicus oleophilus” MY3 (Fig. 4B), a strain closely related to AOA that was dominant in pepper and ginseng rhizospheres (Fig. 1B, D, 2, 3B and 3D; Data set S1). The AOA MnKat, whose active site is predicted to be stable under low H_2_O_2_ levels compared with the heme catalase (63), may provide an evolutionary advantage at low H_2_O_2_ levels (< 3 µM), which can completely inhibit the nitrification activity of catalase-negative AOA (64, 65). Based on the documented selection of MnKat-encoding AOA in rhizospheres of pepper and ginseng plants, as well as the experimental confirmation of catalase activity in a related AOA isolate, it is tempting to speculate that resistance to H_2_O_2_ is one of the important factors shaping AOA communities in rhizospheres. Consistently, we observed that the copy number ratios of the AOA MnKat gene to the *amoA* gene were significantly higher in rhizosphere soils of pepper and ginseng plants than in bulk soils (Fig. 5B and D). The dominance of AOA MnKat gene sequences closely related to “*Ca.* Nitrosocosmicus” in rhizosphere soils (Table S3) corroborated the results of AOA 16S rRNA and *amoA* gene analyses (Fig. 1 and 3).

Soil characteristics (4–9) and host phylogeny (4, 13, 21, 22, 24) are considered important determinants of rhizosphere microbial community composition and function. Even plant genotype-specific microbial communities have been observed in the rhizosphere of some plant species (5, 7). Despite the different life cycles, phylogeny, and geographic locations of the pepper and ginseng plants studied here, distinct AOA communities in the rhizosphere soils relative to the bulk soils were observed, which was attributable to the predominance of the MnKat-containing members of “*Ca.* Nitrosocosmicus” (Fig. 1B, D, 2, 3B and 3D; Data set S1). In this context, it is important to note that the dominant phylotype, C1b.A1 (representing the clones TRC23-30 and TRC23-38), belonging to *Nitrososphaerota* (formerly known as Crenarchaeota), was found to predominantly colonize the roots of tomato (*Solanum lycopersicum* L. in the order Solanales) grown in soil from a Wisconsin field (66). This phylotype is closely related to “*Ca.* Nitrosocosmicus oleophilus” MY3 with 99.7% 16S rRNA gene sequence similarity, suggesting that closely related members of “*Ca.* Nitrosocosmicus” are dominant in various agriculturally important plants and that the enrichment of the AOA in the plant rhizosphere may be widespread, regardless of geographical location and plant phylogeny.

In addition to the *amoA* clade NS-ζ containing members of the genus “*Ca.* Nitrosocosmicus,” the AOA *amoA* gene reads of the clade NS-δ-1 harboring the fosmid clone 54D9 *amoA* sequence were also abundant in the H_2_O_2_-amended soil slurries (Fig. 6D) and the rhizosphere soils of ginseng plants (Fig. 3D), but not in the pepper plants (Fig. 3B). It is yet unknown whether clade NS-δ-1 members have MnKat genes. The prominent increases in the relative abundance of the AOA *amoA* gene reads from clade 54D9 (Fig. 3D and 6D) are in stark contrast with the findings from the analysis of AOA 16S rRNA gene amplicon reads (Fig. 1D and 2A; Fig. 6B). Thus, we cannot rule out the possibility that the PCR primer set used to construct the AOA *amoA* gene amplicon libraries is biased towards clade NS-δ-1 *amoA* genes.

Oxygen supply is crucial for plant roots, not only for cell respiration but also for the formation of reactive oxygen species, including H_2_O_2_. H_2_O_2_ is a ubiquitous metabolic by-product of aerobic unicellular and multicellular organisms (67–70) that plays an important role in developmental and physiological processes in plant roots. H_2_O_2_ is involved in loosening cell walls for cell elongation in roots *via* peroxidase-mediated lignin formation (71, 72) and accumulates in the differentiation zone and the cell wall of root hairs during the formation of fine roots in *Arabidopsis* (*Arabidopsis thaliana* in the order Brassicales) (73). It was observed that H_2_O_2_ production increased in a specific region of fine roots after K^+^ deprivation (74). Similarly, H_2_O_2_ release from seedling roots into the environment has been observed (73, 75–77). Furthermore, mycorrhizae mediated an increase in H_2_O_2_ release from the roots of trifoliate orange to alleviate drought stress (78). Recently, it was proposed that the rhizosphere is a widespread but previously unappreciated hotspot for ROS production, with hydroxyl radicals, which represent ROS species, periodically accumulating up to >2 µM in rice plant rhizosphere soil pore water after 6 h of light exposure (79). Thus, plant roots trigger the release of H_2_O_2_ into their surroundings and thereby chemically shape the rhizosphere habitat. In addition to plant roots, soil microorganisms are known to release ROS (80, 81).

In the soil slurry experiments, we demonstrated that H_2_O_2_ amendment in bulk soils increased the abundance of MnKat-containing AOA in a concentration-dependent manner (Fig. S7). In addition, AOA from the clade “*Ca*. Nitrosocosmicus” became dominant in H_2_O_2_-amended soil slurries (Fig. 6B), and the copy number ratios of AOA MnKat genes to *amoA* genes increased as the concentration of H_2_O_2_ increased (Fig. S7B). The toxic effects of H_2_O_2_ on AOA were previously assessed with group I.1a, where ammonia oxidation was completely inhibited at levels of 0.2–3.0 µM H_2_O_2_ (64, 65). Consistently, the nitrification activity and abundance of AOA decreased in H_2_O_2_-amended soil slurries (Fig. S5 and S6). This might explain the decrease in gross nitrification rates in the rhizosphere (82). Furthermore, “*Ca*. Nitrosocosmicus” MnKat genes were found to be constitutively expressed in pepper plant rhizosphere soils and bulk soils (Fig. S8). Taken together, our results imply that rhizosphere H_2_O_2_ may be an important factor in the selection of MnKat-containing AOA in the plant rhizosphere. Interestingly, metagenomic and metatranscriptomic analyses of the rhizosphere microbial communities of cucumber (*Cucumis sativus* L. in the order Cucurbitales) and wheat (*Triticum turgidum* L. in the order Poales) plants identified the enrichment and expression of prokaryotic catalase genes, which were suggested to be associated with root colonization (83). The dominance of catalase-containing bacterial ASVs in rhizosphere soils of pepper plants over bulk soils (Fig. S4; Data set S2 and S3) observed in this study corresponds to these findings. It is plausible that H_2_O_2_ levels in rhizosphere environments may be inhibitory to catalase-negative microbes such as group I.1a and I.1a-associated AOA. In suspended aquatic environments, the growth of catalase-negative AOA could be supported by coexisting catalase-positive microbes (65, 84, 85). Hence, further study will be needed to reveal if such an interaction exists between catalase-positive microbes and catalase-negative AOA in soil environments. Therefore, we propose that the catalase activity of microorganisms in rhizospheres may serve as a microbial stress response. It may also modulate developmental and physiological processes in plant roots, as well as redox dynamics and biogeochemical processes in soil.

Despite the presence of the MnKat gene in the “*Ca.* Nitrososphaera evergladensis” genome, ASVs related to this AOA were not dominant in the analyzed microbial community in rhizosphere soils (Table S3). Thus, while catalase activity is a very plausible explanation for the selection of members of the genus “*Ca*. Nitrosocosmicus” in the rhizosphere, it should be noted that the genomes of these ammonia-oxidizers also encode various distinct traits that may individually or collectively confer higher rhizosphere fitness compared to other AOA. For example, tolerance to high salinity (51) and acidic pH (86), as well as the ability for biofilm formation (50) observed in “*Ca.* Nitrosocosmicus” members, may support survival in the rhizosphere and/or help establish interactions with plant roots. Furthermore, the higher concentration of ammonia in rhizosphere soils compared to bulk soils (87) might facilitate the competitive success of members of “*Ca.* Nitrosocosmicus,” which possess a lower affinity and lower specific affinity for ammonia than other AOA (88) in the rhizosphere. Thus, more research is needed to determine how catalase activity contributes to the enrichment of “*Ca*. Nitrosocosmicus” members in the rhizospheres of various plants.

The nitrification process, which converts ammonia to nitrite and then to nitrate, strongly affects the availability of nitrogen species for plant roots (89). The available inorganic nitrogen species ratio (ammonium:nitrate) is significant to plant growth by influencing cellular pH maintenance and energy efficiency of nitrogen assimilation in plants (90, 91). Due to their abundance, AOA, especially catalase-containing “*Ca.* Nitrosocosmicus” members, as demonstrated in this study, are considered to be important players mediating the nitrification process in the rhizosphere (59). Song et al. demonstrated that “*Ca*. Nitrosocosmicus oleophilus” MY3 cells colonized the root surface of *Arabidopsis* plants and proposed that volatile compounds emitted by “*Ca*. Nitrosocosmicus oleophilus” MY3 could elicit induced systemic resistance (92). Taken together, the selection of catalase-containing AOA of the genus “*Ca.* Nitrosocosmicus” in the rhizosphere of several agriculturally important plants hints at a previously overlooked AOA-plant interaction. Our understanding of AOA-plant interactions in the rhizosphere is still in its infancy, and this study highlights an important clade of AOA with already available cultured representatives for further mechanistic analyses in this crucial research field.

## MATERIALS AND METHODS

### Plant cultivation, soil collection, and analysis

Soil samples from the bulk and rhizosphere of pepper (*Capsicum annuum* L., Solanaceae) and ginseng (*Panax ginseng* C.A. Meyer, Araliaceae) plants were collected to investigate the plant-root-associated prokaryotic communities. The pepper plants were grown in sandy loam soil under a rain shelter for 6 months (March to September 2017), with the addition of ammonium sulfate (55 kg N ha^−1^) as a nitrogen fertilizer once before transplanting. After transplanting, the average temperature in the rain shelter was kept at 25 ± 5°C, and the soil was watered daily as needed to keep soil moisture above 20% at 30 cm soil depth. A total of 25 soil samples were collected: bulk soil (*n* = 5) at day 0, and both bulk and rhizosphere soils (*n* = 5 each) at 60 days (vegetative phase) and 90 days (reproductive phase). The rhizosphere soil samples were collected as soil tightly adhering to plant roots. Five bulk soil subsamples were collected at 15 cm soil depth and 40 cm away from the plants and then mixed. The soil samples were stored at −80°C until DNA extraction. The locations and general properties of bulk soil are presented in Table S4.

The ginseng plants were grown for 6 years (2012–2018) after transplanting 1-year-old seedlings (Table S4) to a sandy loam soil field. The Ginseng Good Agricultural Practices Scheme from the National Institute of Horticultural and Herbal Science of the Rural Development Administration (Republic of Korea) was followed for pre-planting treatment, pest management, watering, and fertilization. During the cultivation period, a total of 29 soil samples were collected: bulk soils (*n* = 3 for each age group) and rhizosphere soils (*n* = 4, 7, and 9 for 2-, 4-, and 6-year-old ginseng plants, respectively). The collection of soil samples and analysis of general properties of soils were conducted in the same manner as described above for pepper plants.

### DNA extraction and quantification of AOA *amoA* and MnKat genes

Genomic DNA was extracted from each 0.25  g soil sample using the Exgene Soil DNA mini extraction kit (GeneAll Biotechnology Co. Ltd., Republic of Korea) according to the manufacturer’s instructions. The DNA concentration and purity were measured using a Nanodrop ND-1000 spectrophotometer (NanoDrop Technologies, USA). To check for possible quantitative PCR (qPCR) or PCR inhibition, genomic DNA from *Methylacidiphilum caldifontis* IT6 (10^6^ gene copy numbers per reaction) (93) was spiked as an internal positive control (94) into serially diluted template DNA (0.25–20 ng) extracted from soils of the pepper and ginseng plants. Strain IT6-specific *pmoA1* gene primer pair was used for qPCR detection. The quantitative results of the spiked control within the template or pure water were assessed by comparing the Ct values of the *pmoA1* gene. No significant PCR inhibition was observed in the serially diluted template DNA (0.25–20 ng), and thus, we used<10 ng DNA for further qPCR analysis.

The copy numbers of the AOA *amoA* gene were assessed using the CrenamoA104F/CrenamoA616R primer pairs (see Table S5). The primer pair for quantifying the AOA MnKat gene (aoa-MnKat200F: 5′-GAAGAGATRGGWCATGTWGA-3′, aoa-MnKat480R: 5′-CCTGTMGCYTCAAGCATDA-3′) was newly designed using MnKat gene sequences retrieved from the genomes of “*Ca*. Nitrosocosmicus oleophilus” MY3 (GCA_000802205.2), “*Ca*. Nitrosocosmicus arcticus” Kfb (GCA_007826885.1), “*Ca*. Nitrosocosmicus hydrocola” G61 (GCF_001870125.1), and “*Ca*. Nitrososphaera evergladensis” SR1 (GCA_000730285.1). 1–10  ng of sample DNA was used for qPCR using a MiniOpticon qPCR detection system (Bio-Rad Laboratories, Hercules, USA). The iQ SYBR Green Supermix (Bio-Rad Laboratories, USA) and PCR primer pairs were used for PCR amplification. AOA *amoA* gene was amplified via the following steps: 95°C for 3 min; followed by 40 cycles at 95°C for 45 s, 55°C for 45 s, 72°C for 45 s; and 72°C for 5 min. AOA MnKat gene was amplified *via* the following steps: 95°C for 3 min; followed by 40 cycles at 95°C for 45 s, 55°C for 45 s, 72°C for 45 s; and 72°C for 5 min. A dilution series of genomic DNA from strain MY3 was included in every qPCR cycle for calibration purposes. Amplification efficiencies ranged between 80% and 94% for all target genes, and qPCR R^2^ calibration values were greater than 0.99.

### Amplicon sequencing and phylogenetic analysis

For the construction of amplicon sequencing libraries, the 16S rRNA gene was amplified using the 515F/926R primer pairs (see Table S6), via the following steps: 95°C for 3 min; followed by 25 cycles at 95°C for 45 s, 50°C for 45 s, 72°C for 90 s; and 72°C for 5 min. AOA *amoA* gene was amplified with the CrenamoA104F/CrenamoA616R primer pairs (see Table S5), via the following steps: 95°C for 3 min; followed by 30 cycles at 95°C for 45 s, 55°C for 45 s, 72°C for 45 s; and 72°C for 5 min. AOA MnKat gene was amplified with the aoa-MnKat200F/aoa-MnKat480R primer pairs via the following steps: 3 min heating step at 95°C; followed by 35 cycles at 95°C for 45 s, 55°C for 45 s, 72°C for 45 s; and 72°C for 5 min. The following index PCR for both gene libraries was conducted with the Nextera XT index kit v2 (Illumina Inc., USA). The PCR product was purified using the Labopass DNA purification kit (Cosmogenetech Inc., Republic of Korea). The sequencing was performed using the Illumina MiSeq (2 × 300 bp) platform (Illumina Inc., USA) at Macrogen Inc. (Republic of Korea). The QIIME2 (v2024.2) pipeline with implemented tools for quality control (Cutadapt) (95) and de-noising, and pair read merging (DADA2) (96) was used to analyze the amplicon sequence data. The primer region was trimmed. After quality plots were generated, the sequences failing to pass an average base call accuracy of 99% (median Phred score of 20) were excluded. Low-quality regions of each sequence were removed during the de-noising step using DADA2 with the following parameters: 16S rRNA gene: --p-trunc-len-f 264 --p-trunc-len-r 168 --p-max-ee-f 2 --p-max-ee-r 4; AOA *amoA* gene: --p-trunc-len-f 280 --p-trunc-len-r 265 --p-max-ee-f 2 --p-max-ee-r 4; AOA MnKat gene: --p-trunc-len-f 188 --p-trunc-len-r 110 --p-max-ee-f 3 --p-max-ee-r 3(94). ASVs were taxonomically classified using qiime feature-classifer and sklearn algorithm against Greengene2 database (v2022.10) for the 16S rRNA gene, the Alves RJE et al. (49) reference data set for the AOA *amoA* gene, and AOA MnKat genes. For further analyses, the 16S rRNA and the AOA *amoA* gene ASV tables were rarefied to even depths of 42,584 and 43,680 reads, respectively.

For phylogenetic analyses, representative full-length sequences of the AOA 16S rRNA, *amoA*, and MnKat genes were obtained from the NCBI database. Alignments of the derived sequences were performed using MAFFT (v7.453) (97). A maximum likelihood phylogenetic tree was constructed with IQ-TREE (v1.6.12) (48).

### Catalase activity assays

Three strains of AOA (“*Ca*. Nitrosocosmicus oleophilus” MY3, *Nitrosoarchaeum koreense* MY1, and *Nitrososphaera viennensis* EN76) and one strain of AOB (*Nitrosomonas europaea* ATCC 19718) were grown under optimal conditions (98). After oxidizing 1 mM ammonia, the cells were harvested by centrifugation (5,000 × *g* for 20 min at 25°C). The harvested cells were washed three times using a basal artificial freshwater medium (AFM) (98), which is devoid of ammonia, trace elements, and pyruvate. Subsequently, 2 mL aliquots of cell suspension were filled into a respiration chamber fitted with contactless oxygen sensor spots (OXSP5, PyroScience, Germany) and maintained for 10–20  min before H_2_O_2_ injection. The FireSting fiber-optical oxygen meter FSO2-1 (PyroScience, Germany) operation and two‐point calibration followed the manufacturer’s instructions. The reaction chambers were set at the optimal growth temperature for each strain (98) in a water bath (NB-304, N-BIOTEK, Republic of Korea) and stirred with a magnetic stirrer (MIXdrive 1 XS, 2mag AG, Germany) at 1,000 rpm.

### Soil slurry experiment

For soil slurry experiments, the bulk soil composited from five subsamples from the pepper plant experiment was sieved (2-mm mesh size) to remove plant debris and stones. Aliquots of the fresh bulk soil (0.25 g) were incubated in 25 cm^3^ cell culture flasks (SPL Life Sciences, Republic of Korea) containing 10 mL of AFM. The medium contained NH_4_Cl (1.5 mM), NaHCO_3_ (2 mM), and MES buffer (pH 6.5; 3 mM). Allylthiourea (50 µM), an inhibitor of AOB growth (99), was used to focus on the response of soil AOA to H_2_O_2_ amendment. The cultures were incubated under aerobic conditions in the dark at 30°C in a static incubator with intermittent mixing. During the experiment, H_2_O_2_ was amended twice daily to reach final concentrations of 0–30 µM. Nitrite and nitrate were measured using spectrophotometric methods as previously described (98) for the estimation of nitrification activity. For DNA extraction, soil aliquots were obtained from the slurry samples by centrifugation (8,000 × *g* for 20 min at 25°C) and were stored at −80°C.

To estimate the biotic and abiotic decomposition rates of H_2_O_2_ in soil slurries, H_2_O_2_ concentrations in fresh bulk soils and autoclaved soil slurries were measured using ISO-HPO-100 (WPI, UK) amperometric sensors, with an Apollo 4000 System (WPI, UK). For H_2_O_2_ measurements, soil slurries were stirred using an MS-500 magnetic stirrer (Duksan General Science Co., Republic of Korea) at 1,500 rpm. The electrode was calibrated against freshly prepared H_2_O_2_ solutions in the range of 0–10 µM in AFM at 25°C.

### AOA MnKat gene expression assay

Total RNA was extracted from each 2 g of bulk and rhizosphere soil samples (bulk soil: *n* = 5, rhizosphere soil: *n* = 5, from 90-day-old pepper plants) using the RNeasy PowerSoil Total RNA Kit (Qiagen, USA) according to the manufacturer’s instructions. RNA was eluted in 100 µL of RNase-free water. After removing the DNA from the eluent by treatment with DNase, a SuperScript IV VILO ezDNAse kit (Thermo Fisher Scientific, USA) was used for cDNA synthesis. The concentrations of RNA and cDNA were determined using a Qubit 4 fluorometer (Thermo Fisher Scientific, USA).

The relative expression of the AOA MnKat gene in bulk and rhizosphere soils was estimated using a “*Ca*. Nitrosocosmicus” clade-specific housekeeping gene, *rpoB*, and AOA *amoA* gene (see Table S5). The primer pair for quantifying the *rpoB* gene (nsc-rpoB2884F: 5′-TAYGGWTTYAAGCAYAGTGG-3′, nsc-rpoB3320R 5′-TGAGTTTAAATGTSGCWCC-3′) was newly designed using *rpoB* sequences retrieved from “*Ca.* Nitrosocosmicus” clade-genomes (see Fig. S9). The “*Ca.* Nitrosocosmicus” clade-specific *rpoB* gene was amplified via the following steps: 95°C for 3 min; followed by 40 cycles at 95°C for 45 s, 59°C for 45 s, 72°C for 45 s; and 72°C for 5 min.

### Statistical analysis

All statistical analyses were conducted using the R statistical software (v4.1.2) and R Studio (v2022.02.3). Microbial diversity analysis and data visualization were performed using the R packages phyloseq (v1.26.0) (100), vegan (v2.5–3) (101), and ggplot2 (v3.1.0) (102). Processed amplicon sequence reads were imported using phyloseq (100). NMDS analysis and principal coordinate analysis (PCoA) based on Bray–Curtis dissimilarity metrics were used in Vegan (101) to compare the microbial communities between the samples. The ordination analysis patterns were statistically tested using permutational multivariate analysis of variance (PERMANOVA) and analysis of similarity (ANOSIM) with adonis2 and vegdist, respectively, which are part of the vegan packages in R (101). Indicator species were identified using the indval function of labdsv package (v2.0–1) (103). Samples from the same compartment were treated as a group for indicator species analysis.

## Data Availability

The 16S rRNA, AOA *amoA*, and AOA MnKat gene amplicon sequencing data generated in this study have been deposited in NCBI under the BioProject ID PRJNA905906.

## SUPPLEMENTAL MATERIAL

Supplemental data sets msphere.00821-24-s0001.xlsx Data sets S1 to S4.

Supplemental Information msphere.00821-24-s0002.docx Supplemental tables and figures.

ASM does not own the copyrights to Supplemental Material that may be linked to, or accessed through, an article. The authors have granted ASM a non-exclusive, world-wide license to publish the Supplemental Material files. Please contact the corresponding author directly for reuse.
