# Supplementary material for: “Ca. Nitrosocosmicus” members are the dominant archaea associated with plant rhizospheres
Source: mSphere. 2024 Nov 12;9(12):e00821-24. doi: 10.1128/msphere.00821-24 (PMC11656794; doi:10.1128/msphere.00821-24)
Supplement: Supplemental Information — Supplemental tables and figures. [file msphere.00821-24-s0002.docx]

# Supplementary Information

**“*Ca*. Nitrosocosmicus” members are the dominant archaea associated with plant rhizospheres**

Ui-Ju Lee^1,†^, Joo-Han Gwak^1,†^, Seungyeon Choi^1^, Man-Young Jung^2^, Tae Kwon Lee^3^, Hojin Ryu^1^, Samuel Imisi Awala^1^, Wolfgang Wanek^4,5^, Michael Wagner^5,6,7^, Zhe-Xue Quan^8^ & Sung-Keun Rhee^1^

^1^Department of Biological Sciences and Biotechnology, Chungbuk National University, 1 Chungdae-ro, Seowon-Gu, Cheongju 28644, Republic of Korea. ^2^Department of Science Education, Jeju National University, 102 Jejudaehak-ro, Jeju 63243, Korea. ^3^Department of Environmental Engineering, Yonsei University, Wonju, Republic of Korea. ^4^Division of Terrestrial Ecosystem Research, Center of Microbiology and Environmental Systems Science, University of Vienna, Djerassiplatz 1, A-1030 Vienna, Austria. ^5^Department of Microbiology and Ecosystem Science, Centre for Microbiology and Environmental Systems Science, University of Vienna, Vienna, Austria. ^6^The Comammox Research Platform, University of Vienna, Vienna, Austria. ^7^Center for Microbial Communities, Department of Chemistry and Bioscience, Aalborg University, Aalborg, Denmark. ^8^School of Life Sciences, Fudan University, Shanghai, China.

^†^These authors contributed equally to this work.

*Corresponding author. Email: rhees@chungbuk.ac.kr

This document includes:

Supplementary Results and Discussion

Supplementary Tables S1 to S6

Supplementary Figures S1 to S9

Supplementary References

**Supplementary Results and Discussion**

# Distinct prokaryotic communities in rhizosphere soils compared to bulk soils

Prokaryotic 16S rRNA gene amplicon sequencing data revealed the presence of 44 bacterial and 7 archaeal phyla in the rhizosphere and bulk soils of pepper plants (Table S1 and S2). Among these phyla, the relative abundances of *Pseudomonadota*, *Bacteroidota*, and *Actinomycetota* were significantly higher in rhizosphere soils than in bulk soils during the plant vegetative (60-day-old) and reproductive (90-day-old) growth phases. The measured increase in the relative abundance of these three bacterial phyla in rhizosphere soils of pepper plants (Fig. S1A) was also frequently observed in other plant species (1-6). Interestingly, the relative abundance of *Bacillota* increased in rhizosphere soils only during the reproductive phase (90-day-old). *Acidobacteriota*, *Chloroflexota*, and *Gemmatimonadota*, on the other hand, were detected at lower relative abundances in rhizosphere soils (Fig. S1A and Table S1), regardless of the plant growth phase.

The α-diversity of rhizosphere soils, as measured by the Shannon diversity index, was significantly lower compared to that of bulk soils (Fig. S1C). PCoA analysis also clearly separated bulk and rhizosphere soils based on their 16S rRNA gene profiles (Fig. S1B), which was supported by PERMANOVA (*R^2^* = 0.586, F = 32.553, *p* < 0.001). However, the different plant growth phases less explained the variance in rhizosphere soil microbial composition (*R^2^* = 0.166, F = 2.198, *p* > 0.05).

# Table S1. Relative abundance of prokaryotic phyla in bulk and rhizosphere soils of pepper plants. Mean and standard deviation (%) are based on the results from independent replicates of rhizosphere soil (*n* = 15) collected from individual plants at different growth stages, or from bulk soil (*n* = 10) samples. The replication number of each sample type with different plant growth stages is indicated in parentheses following the sample type, either bulk soil or rhizosphere soil. Archaeal and bacterial phyla are listed in order of their relative abundance.

|  | **0-day-old** |  | **60-day-old** | |  | **90-day-old** | |
| --- | --- | --- | --- | --- | --- | --- | --- |
| **Phylum** | **Bulk soil (5)** |  | **Bulk soil (5)** | **Rhizosphere soil (5)** |  | **Bulk soil (5)** | **Rhizosphere soil (5)** |
| **Archaea** | 2.90 ± 0.51 |  | 3.85 ± 0.51 | 0.59 ± 0.10 |  | 4.07 ± 0.32 | 0.83 ± 0.18 |
| *Nitrososphaerota* | 2.82 ± 0.50 |  | 3.7 ± 0.47 | 0.5 ± 0.18 |  | 3.88 ± 0.32 | 0.81 ± 0.16 |
| *Nanoarchaeota* | 0.01 ± 0.01 |  | 0.03 ± 0.04 | 0.04 ± 0.04 |  | 0.06 ± 0.03 | 0.01 ± 0.01 |
| *Thermoplasmatota* | 0.06 ± 0.01 |  | 0.09 ± 0.02 | 0.02 ± 0.03 |  | 0.13 ± 0.06 | 0.00 ± 0.01 |
| Unknown | 0.00 ± 0.00 |  | 0.01 ± 0.01 | 0.02 ± 0.03 |  | 0.00 ± 0.01 | 0.00 ± 0.00 |
| *Halobacteriota* | 0.00 ± 0.00 |  | 0.01 ± 0.02 | 0.00 ± 0.00 |  | 0.00 ± 0.01 | 0.00 ± 0.00 |
| *Ca. Iainarchaeota* | 0.00 ± 0.00 |  | 0.00 ± 0.00 | 0.00 ± 0.00 |  | 0.01 ± 0.01 | 0.00 ± 0.00 |
| *Thermoproteota* | 0.00 ± 0.00 |  | 0.00 ± 0.00 | 0.00 ± 0.00 |  | 0.00 ± 0.00 | 0.00 ± 0.00 |
| **Bacteria** | 96.91 ± 0.62 |  | 96.05 ± 0.58 | 99.23 ± 0.15 |  | 95.9 ± 0.33 | 99.01 ± 0.18 |
| *Pseudomonadota* | 30.67 ± 2.08 |  | 28.24 ± 0.48 | 45.48 ± 6.65 |  | 26.36 ± 1.56 | 40.44 ± 2.57 |
| *Bacteroidota* | 8.99 ± 1.1 |  | 9.75 ± 1.76 | 32.62 ± 4.10 |  | 9.31 ± 1.94 | 28.16 ± 4.97 |
| *Actinomycetota* | 4.37 ± 0.39 |  | 3.96 ± 0.37 | 5.96 ± 2.14 |  | 3.45 ± 0.48 | 7.24 ± 1.26 |
| *Planctomycetota* | 6.88 ± 0.61 |  | 7.16 ± 0.50 | 3.07 ± 0.72 |  | 7.35 ± 0.81 | 3.39 ± 0.94 |
| *Myxococcota* | 2.3 ± 0.29 |  | 2.38 ± 0.17 | 2.35 ± 0.98 |  | 2.19 ± 0.13 | 1.98 ± 0.68 |
| Verrucomicrobiota | 3.55 ± 0.41 |  | 3.18 ± 0.62 | 2.06 ± 0.29 |  | 3.33 ± 0.58 | 1.37 ± 0.36 |
| Unknown | 5.44 ± 0.62 |  | 6.02 ± 0.4 | 2.04 ± 0.26 |  | 6.85 ± 0.67 | 1.73 ± 0.24 |
| *Chloroflexota* | 4.92 ± 0.71 |  | 6.42 ± 0.97 | 1.56 ± 0.35 |  | 6.65 ± 1.00 | 1.62 ± 0.72 |
| *Gemmatimonadota* | 4.99 ± 0.14 |  | 5.19 ± 0.35 | 1.27 ± 0.51 |  | 5.8 ± 0.37 | 1.26 ± 0.28 |
| *Acidobacteriota* | 14.6 ± 0.97 |  | 13.59 ± 1.65 | 1.13 ± 0.22 |  | 13.96 ± 0.94 | 1.92 ± 0.76 |
| *Bacillota*_D | 2.61 ± 0.54 |  | 2.26 ± 0.45 | 0.8 ± 0.67 |  | 2.16 ± 0.5 | 8.21 ± 3.07 |
| *Cyanobacteriota* | 0.25 ± 0.09 |  | 0.29 ± 0.10 | 0.38 ± 0.29 |  | 0.13 ± 0.03 | 0.77 ± 0.72 |
| *Nitrospirota*_A_437815 | 1.08 ± 0.13 |  | 1.01 ± 0.12 | 0.3 ± 0.14 |  | 1.02 ± 0.25 | 0.44 ± 0.16 |
| *Bdellovibrionota*_E | 0.38 ± 0.15 |  | 0.16 ± 0.04 | 0.14 ± 0.02 |  | 0.2 ± 0.08 | 0.06 ± 0.04 |
| *Armatimonadota* | 0.27 ± 0.06 |  | 0.27 ± 0.09 | 0.1 ± 0.04 |  | 0.27 ± 0.10 | 0.09 ± 0.02 |
| *Desulfobacterota*_B | 2.19 ± 0.15 |  | 2.41 ± 0.06 | 0.08 ± 0.04 |  | 2.69 ± 0.31 | 0.21 ± 0.12 |
| *Ca*. Methylomirabilota | 1.47 ± 0.11 |  | 1.55 ± 0.25 | 0.05 ± 0.03 |  | 1.62 ± 0.29 | 0.15 ± 0.08 |
| *Fibrobacterota* | 0.04 ± 0.02 |  | 0.02 ± 0.01 | 0.02 ± 0.01 |  | 0.01 ± 0.01 | 0.00 ± 0.00 |
| *Desulfobacterota*_D | 0.13 ± 0.02 |  | 0.16 ± 0.03 | 0.02 ± 0.02 |  | 0.15 ± 0.02 | 0.02 ± 0.02 |
| *Ca*. Eisenbacteria | 1.14 ± 0.14 |  | 1.12 ± 0.13 | 0.02 ± 0.02 |  | 1.37 ± 0.18 | 0.05 ± 0.04 |
| *Ca*. Hydrogenedentota | 0.05 ± 0.02 |  | 0.08 ± 0.04 | 0.01 ± 0.01 |  | 0.10 ± 0.02 | 0.01 ± 0.01 |
| *Elusimicrobiota* | 0.28 ± 0.10 |  | 0.3 ± 0.03 | 0.01 ± 0.01 |  | 0.27 ± 0.03 | 0.02 ± 0.01 |
| *Chlamydiota* | 0.00 ± 0.00 |  | 0.00 ± 0.00 | 0.01 ± 0.01 |  | 0.01 ± 0.01 | 0.00 ± 0.00 |
| *Bacillota*_A | 0.15 ± 0.04 |  | 0.09 ± 0.02 | 0.01 ± 0.00 |  | 0.11 ± 0.02 | 0.01 ± 0.01 |
| *Ca*. Sumerlaeota | 0.01 ± 0.01 |  | 0.02 ± 0.01 | 0.01 ± 0.01 |  | 0.01 ± 0.01 | 0.01 ± 0.01 |
| *Spirochaetota* | 0.03 ± 0.02 |  | 0.04 ± 0.03 | 0.01 ± 0.01 |  | 0.02 ± 0.01 | 0.01 ± 0.01 |
| *Ca*. Omnitrophota | 0.02 ± 0.02 |  | 0.06 ± 0.03 | 0.00 ± 0.00 |  | 0.08 ± 0.02 | 0.00 ± 0.01 |
| *Desulfobacterota*_E | 0.00 ± 0.00 |  | 0.00 ± 0.00 | 0.00 ± 0.01 |  | 0.00 ± 0.00 | 0.02 ± 0.04 |
| *Desulfobacterota*_G_459546 | 0.01 ± 0.01 |  | 0.01 ± 0.00 | 0.00 ± 0.00 |  | 0.00 ± 0.00 | 0.00 ± 0.01 |
| *Deinococcota* | 0.00 ± 0.01 |  | 0.01 ± 0.02 | 0.00 ± 0.00 |  | 0.05 ± 0.06 | 0.00 ± 0.00 |
| *Desulfobacterota*_I | 0.03 ± 0.01 |  | 0.03 ± 0.01 | 0.00 ± 0.00 |  | 0.04 ± 0.01 | 0.00 ± 0.00 |
| candidate division FCPU426 | 0.08 ± 0.04 |  | 0.09 ± 0.03 | 0.00 ± 0.00 |  | 0.11 ± 0.03 | 0.00 ± 0.00 |
| *Bacillota*_F | 0.01 ± 0.00 |  | 0.00 ± 0.00 | 0.00 ± 0.00 |  | 0.01 ± 0.01 | 0.00 ± 0.00 |
| *Ca*. Zixiibacteriota | 0.03 ± 0.01 |  | 0.12 ± 0.06 | 0.00 ± 0.00 |  | 0.16 ± 0.06 | 0.00 ± 0.00 |
| *Atribacterota* | 0.00 ± 0.00 |  | 0.00 ± 0.01 | 0.00 ± 0.00 |  | 0.00 ± 0.00 | 0.00 ± 0.00 |
| *Desulfobacterota*_G_459544 | 0.00 ± 0.00 |  | 0.00 ± 0.01 | 0.00 ± 0.00 |  | 0.00 ± 0.00 | 0.00 ± 0.00 |
| *Ca*. Dormibacterota | 0.00 ± 0.00 |  | 0.01 ± 0.00 | 0.00 ± 0.00 |  | 0.00 ± 0.00 | 0.00 ± 0.00 |
| *Bacillota*_B_370527 | 0.00 ± 0.01 |  | 0.00 ± 0.00 | 0.00 ± 0.00 |  | 0.00 ± 0.00 | 0.00 ± 0.00 |
| *Bacillota*_B_370533 | 0.00 ± 0.01 |  | 0.00 ± 0.00 | 0.00 ± 0.00 |  | 0.00 ± 0.00 | 0.00 ± 0.00 |
| *Bacillota*_B_370539 | 0.03 ± 0.04 |  | 0.01 ± 0.01 | 0.00 ± 0.00 |  | 0.02 ± 0.02 | 0.00 ± 0.00 |
| *Bacillota*_E | 0.06 ± 0.03 |  | 0.03 ± 0.02 | 0.00 ± 0.00 |  | 0.03 ± 0.03 | 0.00 ± 0.00 |
| *Bacillota*_G | 0.01 ± 0.00 |  | 0.01 ± 0.02 | 0.00 ± 0.00 |  | 0.02 ± 0.01 | 0.00 ± 0.00 |
| *Ca*. Krumholzibacteriota | 0.02 ± 0.02 |  | 0.07 ± 0.06 | 0.00 ± 0.00 |  | 0.08 ± 0.04 | 0.01 ± 0.01 |
| *Myxococcota*_A_437813 | 0.02 ± 0.02 |  | 0.01 ± 0.01 | 0.00 ± 0.00 |  | 0.01 ± 0.01 | 0.00 ± 0.00 |

# Table S2. Relative abundance of prokaryotic phyla in bulk and rhizosphere soils of pepper plants. Mean and standard deviation (%) are based on the results from independent replicates of rhizosphere soil (*n* = 20) collected from individual plants at different growth stages, or from bulk soil (*n* = 9) samples. The replication number of each sample type with different plant growth stages is indicated in parentheses following the sample type, either bulk soil or rhizosphere soil. Archaeal and bacterial phyla are listed in order of their relative abundance.

|  | **2-year-old** | |  | **4-year-old** | |  | **6-year-old** | |
| --- | --- | --- | --- | --- | --- | --- | --- | --- |
| **Phylum** | **Bulk soil (3)** | **Rhizosphere soil (4)** |  | **Bulk soil (3)** | **Rhizosphere soil (7)** |  | **Bulk soil (3)** | **Rhizosphere soil (9)** |
| **Archaea** | 1.32 ± 0.65 | 1.53 ± 1.13 |  | 1.67 ± 0.70 | 0.37 ± 0.14 |  | 1.89 ± 0.70 | 0.50 ± 0.64 |
| *Nitrososphaerota* | 1.3 ± 0.66 | 1.47 ± 1.07 |  | 1.47 ± 0.46 | 0.37 ± 0.14 |  | 1.36 ± 0.19 | 0.47 ± 0.56 |
| *Thermoplasmatota* | 0.00 ± 0.00 | 0.04 ± 0.04 |  | 0.10 ± 0.15 | 0.00 ± 0.00 |  | 0.39 ± 0.46 | 0.02 ± 0.07 |
| *Nanoarchaeota* | 0.00 ± 0.00 | 0.01 ± 0.02 |  | 0.00 ± 0.00 | 0.00 ± 0.00 |  | 0.07 ± 0.07 | 0.00 ± 0.01 |
| *Halobacteriota* | 0.00 ± 0.00 | 0.01 ± 0.01 |  | 0.00 ± 0.00 | 0.00 ± 0.00 |  | 0.00 ± 0.00 | 0.00 ± 0.00 |
| *Methanobacteriota* | 0.00 ± 0.00 | 0.01 ± 0.01 |  | 0.00 ± 0.00 | 0.00 ± 0.00 |  | 0.00 ± 0.00 | 0.00 ± 0.00 |
| *Thermoproteota* | 0.00 ± 0.00 | 0.00 ± 0.00 |  | 0.00 ± 0.00 | 0.00 ± 0.00 |  | 0.00 ± 0.00 | 0.00 ± 0.00 |
| Unknown | 0.01 ± 0.02 | 0.00 ± 0.00 |  | 0.09 ± 0.12 | 0.00 ± 0.00 |  | 0.07 ± 0.03 | 0.00 ± 0.00 |
| **Bacteria** | 98.61 ± 0.61 | 98.46 ± 1.14 |  | 98.31 ± 0.68 | 99.6 ± 0.13 |  | 98.08 ± 0.69 | 99.43 ± 0.61 |
| *Pseudomonadota* | 37.9 ± 5.36 | 47.12 ± 12.51 |  | 42.31 ± 6.19 | 64.29 ± 3.03 |  | 38.26 ± 6.25 | 56.25 ± 7.44 |
| *Bacteroidota* | 2.47 ± 0.23 | 10.57 ± 4.07 |  | 4.53 ± 3.24 | 10.87 ± 2.85 |  | 2.88 ± 0.35 | 10.87 ± 3.24 |
| *Actinomycetota* | 10.03 ± 0.41 | 8.84 ± 2.11 |  | 13.49 ± 5.02 | 9.90 ± 1.92 |  | 12.5 ± 3.31 | 10.05 ± 2.52 |
| *Acidobacteriota* | 16.39 ± 2.13 | 6.86 ± 5.10 |  | 13.74 ± 2.31 | 1.64 ± 0.33 |  | 14.31 ± 2.18 | 3.81 ± 3.77 |
| *Chloroflexota* | 5.47 ± 1.07 | 6.00 ± 3.81 |  | 3.67 ± 0.75 | 1.07 ± 0.46 |  | 5.08 ± 1.36 | 2.12 ± 2.08 |
| *Planctomycetota* | 5.04 ± 1.03 | 4.93 ± 2.00 |  | 3.59 ± 0.61 | 4.97 ± 1.52 |  | 4.77 ± 0.73 | 6.01 ± 1.28 |
| Unknown | 8.28 ± 0.73 | 3.17 ± 1.55 |  | 6.61 ± 1.17 | 1.38 ± 0.21 |  | 5.23 ± 0.92 | 2.47 ± 1.14 |
| Verrucomicrobiota | 1.52 ± 0.61 | 2.61 ± 0.58 |  | 2.73 ± 1.37 | 1.82 ± 0.13 |  | 3.18 ± 0.87 | 1.77 ± 0.55 |
| *Gemmatimonadota* | 1.95 ± 0.48 | 1.99 ± 1.53 |  | 3.04 ± 1.54 | 0.52 ± 0.27 |  | 4.71 ± 0.53 | 1.32 ± 0.86 |
| *Myxococcota* | 1.02 ± 0.6 | 1.51 ± 0.43 |  | 0.73 ± 0.15 | 1.82 ± 0.20 |  | 1.17 ± 0.28 | 1.48 ± 0.45 |
| *Bacillota*_D | 2.19 ± 0.47 | 1.01 ± 0.82 |  | 0.73 ± 0.25 | 0.35 ± 0.42 |  | 1.65 ± 0.94 | 1.47 ± 1.86 |
| *Desulfobacterota*_B | 0.69 ± 0.12 | 0.74 ± 0.58 |  | 0.52 ± 0.26 | 0.19 ± 0.14 |  | 0.95 ± 0.31 | 0.46 ± 0.33 |
| Ca. Eisenbacteria | 0.00 ± 0.00 | 0.65 ± 0.58 |  | 0.11 ± 0.15 | 0.02 ± 0.02 |  | 0.59 ± 0.35 | 0.10 ± 0.19 |
| *Nitrospirota*_A_437815 | 0.12 ± 0.02 | 0.49 ± 0.33 |  | 0.35 ± 0.22 | 0.08 ± 0.04 |  | 0.68 ± 0.01 | 0.21 ± 0.30 |
| *Ca*. Methylomirabilota | 0.00 ± 0.00 | 0.37 ± 0.37 |  | 0.04 ± 0.04 | 0.08 ± 0.03 |  | 0.32 ± 0.21 | 0.18 ± 0.41 |
| *Armatimonadota* | 0.4 ± 0.18 | 0.34 ± 0.19 |  | 0.38 ± 0.19 | 0.02 ± 0.02 |  | 0.54 ± 0.25 | 0.09 ± 0.11 |
| *Bacillota*_A | 1.13 ± 0.37 | 0.26 ± 0.21 |  | 0.17 ± 0.21 | 0.12 ± 0.03 |  | 0.11 ± 0.09 | 0.05 ± 0.07 |
| *Bdellovibrionota*_E | 0.62 ± 0.12 | 0.25 ± 0.17 |  | 0.18 ± 0.08 | 0.14 ± 0.03 |  | 0.18 ± 0.02 | 0.12 ± 0.10 |
| *Desulfobacterota*_G_459546 | 0.03 ± 0.02 | 0.17 ± 0.24 |  | 0.01 ± 0.01 | 0.01 ± 0.01 |  | 0.07 ± 0.06 | 0.02 ± 0.04 |
| *Cyanobacteriota* | 0.15 ± 0.03 | 0.17 ± 0.19 |  | 0.19 ± 0.14 | 0.26 ± 0.06 |  | 0.26 ± 0.21 | 0.41 ± 0.23 |
| *Spirochaetota* | 0.00 ± 0.00 | 0.08 ± 0.06 |  | 0.00 ± 0.00 | 0.05 ± 0.02 |  | 0.01 ± 0.01 | 0.02 ± 0.01 |
| *Elusimicrobiota* | 0.34 ± 0.10 | 0.06 ± 0.06 |  | 0.30 ± 0.14 | 0.01 ± 0.02 |  | 0.26 ± 0.18 | 0.05 ± 0.04 |
| *Desulfobacterota*_E | 0.00 ± 0.00 | 0.04 ± 0.06 |  | 0.00 ± 0.00 | 0.00 ± 0.00 |  | 0.00 ± 0.00 | 0.00 ± 0.00 |
| *Desulfobacterota*_I | 0.00 ± 0.00 | 0.03 ± 0.04 |  | 0.00 ± 0.00 | 0.00 ± 0.00 |  | 0.00 ± 0.00 | 0.00 ± 0.00 |
| *Fibrobacterota* | 0.02 ± 0.02 | 0.03 ± 0.03 |  | 0.02 ± 0.02 | 0.00 ± 0.00 |  | 0.01 ± 0.01 | 0.00 ± 0.01 |
| *Desulfobacterota*_C | 0.00 ± 0.00 | 0.02 ± 0.03 |  | 0.00 ± 0.00 | 0.00 ± 0.00 |  | 0.00 ± 0.00 | 0.00 ± 0.00 |
| candidate division FCPU426 | 0.20 ± 0.09 | 0.02 ± 0.03 |  | 0.06 ± 0.04 | 0.00 ± 0.00 |  | 0.04 ± 0.03 | 0.00 ± 0.01 |
| *Bacillota*_B_370539 | 0.00 ± 0.00 | 0.02 ± 0.02 |  | 0.00 ± 0.00 | 0.00 ± 0.00 |  | 0.01 ± 0.01 | 0.00 ± 0.00 |
| *Ca*. Omnitrophota | 0.14 ± 0.19 | 0.02 ± 0.03 |  | 0.03 ± 0.05 | 0.00 ± 0.00 |  | 0.10 ± 0.12 | 0.00 ± 0.01 |
| *Ca*. Sumerlaeota | 0.00 ± 0.00 | 0.02 ± 0.02 |  | 0.01 ± 0.02 | 0.00 ± 0.00 |  | 0.00 ± 0.00 | 0.01 ± 0.02 |
| *Ca*. Tectomicrobia | 0.00 ± 0.00 | 0.02 ± 0.03 |  | 0.00 ± 0.00 | 0.00 ± 0.00 |  | 0.00 ± 0.00 | 0.00 ± 0.00 |
| *Chlamydiota* | 0.01 ± 0.01 | 0.01 ± 0.01 |  | 0.01 ± 0.01 | 0.06 ± 0.04 |  | 0.02 ± 0.01 | 0.16 ± 0.11 |
| *Ca*. Hydrogenedentota | 0.00 ± 0.00 | 0.01 ± 0.01 |  | 0.00 ± 0.00 | 0.00 ± 0.00 |  | 0.03 ± 0.02 | 0.00 ± 0.00 |
| *Bacillota*_C | 0.09 ± 0.04 | 0.01 ± 0.01 |  | 0.01 ± 0.01 | 0.00 ± 0.00 |  | 0.01 ± 0.01 | 0.00 ± 0.01 |
| *Myxococcota*_A_437813 | 0.00 ± 0.00 | 0.01 ± 0.01 |  | 0.00 ± 0.00 | 0.01 ± 0.01 |  | 0.03 ± 0.03 | 0.02 ± 0.02 |
| *Ca*. Schekmanbacteria | 0.00 ± 0.00 | 0.00 ± 0.01 |  | 0.00 ± 0.00 | 0.00 ± 0.00 |  | 0.01 ± 0.01 | 0.00 ± 0.01 |
| unclassified *Armatimonadota* CSP1-3 | 0.00 ± 0.00 | 0.00 ± 0.01 |  | 0.00 ± 0.00 | 0.00 ± 0.00 |  | 0.00 ± 0.00 | 0.00 ± 0.00 |
| *Bacillota*_E | 0.00 ± 0.00 | 0.00 ± 0.01 |  | 0.00 ± 0.00 | 0.00 ± 0.00 |  | 0.01 ± 0.01 | 0.00 ± 0.00 |
| *Ca*. Zixiibacteriota | 0.00 ± 0.00 | 0.00 ± 0.01 |  | 0.00 ± 0.00 | 0.00 ± 0.00 |  | 0.00 ± 0.00 | 0.00 ± 0.00 |
| *Desulfobacterota*_G_459544 | 0.00 ± 0.00 | 0.00 ± 0.00 |  | 0.00 ± 0.00 | 0.00 ± 0.00 |  | 0.03 ± 0.03 | 0.00 ± 0.01 |
| *Ca*. Dormibacterota | 0.92 ± 0.34 | 0.00 ± 0.00 |  | 0.32 ± 0.11 | 0.00 ± 0.00 |  | 0.06 ± 0.01 | 0.00 ± 0.00 |
| *Bacillota*_G | 0.01 ± 0.01 | 0.00 ± 0.00 |  | 0.00 ± 0.00 | 0.01 ± 0.00 |  | 0.01 ± 0.01 | 0.00 ± 0.01 |
| *Desulfobacterota*_D | 0.00 ± 0.00 | 0.00 ± 0.00 |  | 0.00 ± 0.00 | 0.00 ± 0.00 |  | 0.00 ± 0.00 | 0.00 ± 0.01 |
| *Vulcanimicrobiota* | 1.59 ± 0.07 | 0.00 ± 0.00 |  | 0.46 ± 0.34 | 0.00 ± 0.01 |  | 0.05 ± 0.03 | 0.04 ± 0.03 |

# Table S3. Composition of AOA MnKat gene sequences amplified from soil samples using PCR primers designed in this study. Mean and standard deviation (%) from biological replicates are shown. See Dataset S4 and Fig. S3 for the information on the ASVs of the MnKat gene sequences and the phylogeny of the ASVs, respectively.

|  | **Pepper plant** | | | |  | **Ginseng plant** | | | |
| --- | --- | --- | --- | --- | --- | --- | --- | --- | --- |
|  | **60-day-old** | | **90-day-old** | |  | **4-year-old** | | **6-year-old** | |
| **Clade** | **Bulk soil (4)** | **Rhizosphere soil (5)** | **Bulk soil (5)** | **Rhizosphere soil (5)** |  | **Bulk soil (3)** | **Rhizosphere soil (6)** | **Bulk soil (3)** | **Rhizosphere soil (8)** |
| “*Ca.* N. oleophilus” MY3-like | 94.42 ± 3.16 | 97.09 ± 2.71 | 96.36 ± 3.55 | 95.35 ± 4.13 |  | 65.78 ± 42.78 | 99.61 ± 0.57 | 67.23 ± 44.32 | 99.78 ± 0.33 |
| “*Ca.* N. everglandensis” SR1-like | 1.96 ± 2.34 | 0.72 ± 1.31 | 1.00 ± 2.00 | 2.51 ± 4.55 |  | 31.54 ± 42.60 | 0.3 ± 0.4 | 32.77 ± 44.32 | 0.22 ± 0.33 |
| *“Ca.* N. gargensis”  Ga9.2-like | 3.62 ± 1.84 | 2.19 ± 1.67 | 2.64 ± 3.62 | 2.14 ± 0.51 |  | 2.32 ± 2.20 | 0.05 ± 0.11 | – | – |
| Unclassified *Nitrososphaerota* | – | – | – | – |  | 0.36 ± 0.51 | 0.04 ± 0.09 | – | – |

The number of replicates is shown in parenthesis following the sample name.

# Table S4. Properties of agricultural station soils used for cultivating pepper and ginseng plants.

|  | **Pepper** | | |  | **Ginseng** | | | | |
| --- | --- | --- | --- | --- | --- | --- | --- | --- | --- |
| **Variable** | **60-day-old** |  | **90-day-old** |  | **2-year-old** |  | **4-year-old** |  | **6-year-old** |
| **Coordinates** | 36° 30' 26. 5" N  126° 55' 58. 6" E | | |  | 36° 56' 26. 0" N  127° 45' 03. 0" E | | | | |
| **Soil texture** | Sandy loam | | |  | Sandy loam | | | | |
| **Sand content (%)** | 57.2 | | |  | 51.7 | | | | |
| **Silt content (%)** | 27.6 | | |  | 44.0 | | | | |
| **Clay content (%)** | 15.2 | | |  | 4.3 | | | | |
| **pH** | 6.3 |  | 6.7 |  | 6.6 |  | 5.8 |  | 5.7 |
| **Electrical conductivity (dS m ^-1^)** | 0.7 |  | 1.1 |  | 0.39 |  | 0.69 |  | 0.52 |
| **Organic Matter (g kg ^-1^)** | 28.8 |  | 29.0 |  | 24.5 |  | 16.1 |  | 17.7 |
| **Available P_2_O_5_ (mg kg ^-1^)** | 662 |  | 601 |  | 94 |  | 28 |  | 18 |
| **Total Nitrogen (g kg ^-1^)** | 1.3 |  | 1.1 |  | 0.3 |  | 0.1 |  | 0.1 |
| **NH₄⁺-N (mg kg ^-1^)** | 13 |  | 18 |  | 18 |  | 2.5 |  | 2.2 |
| **NO_3_⁻-N (mg kg ^-1^)** | 17 |  | 62 |  | 40 |  | 29.5 |  | 20 |
| **Exchangeable cations** |  |  |  |  |  |  |  |  |  |
| **Potassium (K) (cmol kg ^-1^)** | 0.53 |  | 0.61 |  | 0.4 |  | 0.47 |  | 0.3 |
| **Calcium (Ca) (cmol kg ^-1^)** | 8.6 |  | 8.3 |  | 6.05 |  | 7.17 |  | 5.09 |
| **Magnesium (Mg) (cmol kg ^-1^)** | 2.0 |  | 2.6 |  | 2.13 |  | 2.91 |  | 2.62 |
| **Sodium (Na) (cmol kg ^-1^)** | 0.21 |  | 0.11 |  | 0.1 |  | 0.3 |  | 0.17 |

# Table S5. Primer set used for AOA *amoA* gene amplicon sequencing library and qPCR. Because the majority of AOA *amoA* gene sequences in public databases are amplicon library sequences that used ‘Crenamo’ primers, the coverage of the primer pair was calculated using AOA *amoA* gene sequences retrieved from high-quality AOA genomes in GTDB (R207) (7) and NCBI nr databases.

|  | **% Coverage in reference sequence data^a,b^** | | | | | | | | | | | | | | | |  |  |
| --- | --- | --- | --- | --- | --- | --- | --- | --- | --- | --- | --- | --- | --- | --- | --- | --- | --- | --- |
| **Primer name** | **NP-γ** | **NP-θ** | **NP-η** | **NP-δ** | **NP-ε** | **NP-α** | **NT-α** | **NT-β** | **NS-γ** | **NS-β** | **NS-ζ** | **NS-ε** | **NS-Is-1^c^** | **NS-δ** | **NS-α** | **NC** | **Sequence (5′ to 3′)** | **Reference** |
| CrenamoA104F | 74.1  /84.5 (58) | 22.2  /55.6 (9) | 46.2  /84.6 (13) | 0  /100 (2) | 44.4  /100 (9) | 0  /11.1 (9) | 43.8  /100 (16) | 0  /100 (1) | 100  /100 (4) | 0  /100 (4) | 90.9  /100 (11) | 0  /100 (1) | 100  /100 (1) | 100  /100 (7) | 100  /100 (4) | 0  /0 (7) | GCAGGWGAYTAYATHTTCTA | [Tourna et al (2011)^8^](#_ENREF_8) |
| CrenamoA616R |  |  |  |  |  |  |  |  |  |  |  |  |  |  |  |  | GCCATCCATCTRTADGTCCA |  |

^a^The percentage of sequences that do not contain mismatches/ the percentage of sequences that do not contain a mismatch and those that contain one mismatch in the last four positions near the 3′ end of the primer.

^b^The number in parentheses is the number of reference genome sequences in the particular taxonomic group.

^c^NS-Incertae_sedis-1.

# Table S6. Primer set used for the preparation of 16S rRNA gene amplicon sequencing library. The coverage of the primer pair was calculated using the SILVA TestPrime tool with the SSU database (r138.1) (9).

|  | **% Coverage in reference sequence data^a,b^** | | |  |  |
| --- | --- | --- | --- | --- | --- |
| **Primer name** | **Archaea** | ***Nitrososphaerota*** | **Bacteria** | **Sequence (5′ to 3′)** | **Reference** |
| 515F | 81.0/90.1  (19,976) | 83.4/88.9  (7,547) | 84.5/92.2  (381,528) | GTGYCAGCMGCCGCGGTAA | [Osbrun et al (2011)^10^](#_ENREF_10) |
| 926R |  |  |  | CCGYCAATTYMTTTRAGTTT |  |

^a^The percentage of sequences that do not contain mismatches/ the percentage of sequences that do not contain a mismatch and those that contain one mismatch in the last four positions near the 3′ end of the primer.

^b^The number in parentheses is the number of reference sequences in the particular taxonomic group.

# Fig. S1: Comparison of the prokaryotic communities between bulk and rhizosphere soils of pepper plants.

**A** Relative abundance (%) of the top 14 most abundant phyla detected in prokaryotic 16S rRNA gene profiles (see Table S1). All reads that are mapped to other phyla or that were not classifiable at a phylum level are grouped into the fifteenth category, titled “Others”. A total of 25 samples were collected: bulk soil samples (*n* = 5) at day 0, and bulk (*n* = 5) and rhizosphere soil samples (*n* = 5) at both 60 and 90 days. **B** Principal coordinates analysis (PCoA) plot using Bray–Curtis dissimilarity metrics of prokaryotic communities in bulk and rhizosphere soils, based on prokaryotic 16S rRNA gene profiles. **C** Alpha diversity is measured by the Shannon diversity index based on prokaryotic 16S rRNA gene profiles. Each median value is shown as a line within the boxes. The top and bottom of the boxes represent the 75th and 25th percentiles, respectively. Whiskers represent 1.5 times the interquartile range. Possible outliers are shown as dots. Statistical significance was determined using Student’s t-tests (**, *p* < 0.005; ***, *p* < 0.0005).

A

B

# Fig. S2: Relative abundance of ASVs of ammonia-oxidizing microorganisms in bulk and rhizosphere soils of pepper and ginseng plants.

Relative abundances (% of the total 16S rRNA gene reads) of ammonia-oxidizing microorganisms in bulk (B) and rhizosphere (R) soils, based on 16S rRNA gene profiles of pepper and ginseng plants. In pepper plants, a total of 25 samples were collected: bulk soil samples (*n* = 15) and rhizosphere soil samples (*n* = 10) (**A**). For ginseng plants, a total of 29 samples were collected: bulk soils samples (*n* = 9) and rhizosphere soil samples (*n* = 20) (**B**). Significant differences between ammonia-oxidizing microorganisms are indicated by different letters (One-way ANOVA, Tukey’s test, *p* *<* 0.05).

# Fig. S3: Maximum likelihood phylogenetic tree of MnKat gene

The phylogenetic tree was reconstructed based on nucleic acid sequences of MnKat using IQ-TREE, and the best-fit model (GTR+F+R10) was determined using ModelFinder Plus (11) within IQ-TREE (12). AOA MnKat gene ASVs with more than 96% sequence similarity on nucleotide level were grouped into single cluster and cluster information displayed in Data Set S4. Branch supports for 1,000 replicates were obtained using the ultrafast bootstrap and SH-aLRT tests. MnKat-containing AOAs are emphasized with bold and blue letters. A truncated MnKat-containing AOA is indicated with bold and brown letters. Branch supports ≥ 95% are indicated by black circles.

# Fig. S4: Catalase-positive or -negative 16S rRNA ASVs of prokaryotes between bulk and rhizosphere soils of pepper plants in the reproductive phase (90-day-old).

A total of 131 ASVs were chosen, with a relative abundance greater than 0.2%, and whose average relative abundance changed more than 10-fold (> 3.32 log_2_fold change) between bulk and rhizosphere soils (see Data set S2). The taxonomy of ASVs was identified by VSEARCH using the Greengenes2 database (13). If an ASV was identified at the genus level, it was labeled as “catalase-positive” (red) or “catalase-negative” (blue). If over 50% of the all MAGs assigned from that taxonomy in GTDB contained a catalase gene, as determined through BLAST analysis (Data set S3), or if catalase activity had been confirmed in previous studies, it was classified as “catalase-positive” (red). If neither criterion was met, the ASV was classified as “catalase-negative” (blue). ASVs that could not be assigned to a genus level were classified as "Unknown" (gray). ASVs that are found only in bulk or rhizosphere soils were labeled as bulk soil-specific or rhizosphere soil-specific.

# Fig. S5: Changes in NO_2_^−^ + NO_3_^−^ concentrations during incubation of the soil slurries amended with different H_2_O_2_ concentrations.

Each point represents the mean value, and error bars represent the standard deviation (*n* = 3).

# Fig. S6: Decomposition of H_2_O_2_ in the soil slurries.

Time-course measurements of H_2_O_2_ concentration after spiking H_2_O_2_ into the soil slurry samples. An autoclaved soil slurry sample was used as a negative control for abiotic decomposition

.

*

c

bc

a

b

A

B

# Fig. S7: Abundance of AOA *amoA* and MnKat genes in soil slurries amended with different H_2_O_2_ concentrations.

**A** The copy numbers of AOA *amoA* and MnKat genes in soil slurries with different concentrations of amended-H_2_O_2_. **B** The copy number ratios (%) of AOA MnKat gene to *amoA* gene calculated from (**A**) are shown. Error bars represent the standard deviations (*n* = 5). Significant differences between H_2_O_2_ concentrations are indicated by an asterisk (**A**) and different letters (**B**) (One-way ANOVA, Tukey’s test, *p* *<* 0.05).

# Fig. S8: AOA MnKat gene expression in bulk and rhizosphere soils of the pepper plants.

Relative gene expression of AOA MnKat gene to the expression of a key gene of AOA, i.e., *amoA* and “*Ca.* Nitrosocosmicus” clade-specific housekeeping *rpoB*, in bulk soil samples (B, *n* = 5) and rhizosphere soil samples (R, *n* = 5) of 90-day-old pepper plants.

|  | Forward region (2884) | Reverse region (3320) |
| --- | --- | --- |
| *Ca.* Nitrosocaldus cavascurensis SCU2  *Ca.* Nitrosocaldus islandicus 3F  *Nitrosopumilus piranensis* D3C  *Nitrosopumilus maritimus* SCM1  *Ca*. Nitrosopumilus sp. SW  *Nitrosopumilus cobalaminigenes* HCA1  *Nitrosopumilus oxyclinae* HCE1  *Ca.* Nitrosopumilus sediminis AR2  *Ca.* Nitrosopumilus koreensis AR1  *Ca.* Nitrosomarinus catalina SPOT01  *Nitrosopumilus* sp. Nsub  *Ca.* Nitrosopumilus salaria BD31  *Nitrosopumilus zosterae* NM25  *Nitrosopumilus ureiphilus* PS0  *Nitrosopumilus adriaticus* NF5  *Ca.* Nitrosarchaeum limnium SFB1  *Nitrosarchaeum koreense* MY1  *Ca.* Nitrosopelagicus brevis CN25  *Ca.* Nitrosotenuis aquarius AQ6f  *Ca.* Nitrosotenuis cloacae SAT1  *Ca.* Nitrosotenuis uzonensis N4  *Ca.* Nitrosotenuis sp. DW1  *Ca.* Nitrosotenuis chungbukensis MY2  *Ca.* Nitrosotalea devanaterra Nd1  *Ca.* Nitrosotalea okcheonensis CS  *Ca.* Nitrosotalea sp. FS  *Ca.* Nitrosotalea sinensis Nd2  *Ca.* Nitrosotalea bavarica SBT1  *Nitrososphaera* sp. AFS  *Ca.* Nitrososphaera gargensis Ga9.2  *Ca.* Nitrososphaera evergladensis SR1  *Nitrososphaera viennensis* EN76  *Ca.* Nitrosocosmicus sp. RBC AOA2  *Ca.* Nitrosocosmicus franklandus C13  *Ca.* Nitrosocosmicus sp. WA-bin7  *Ca.* Nitrosocosmicus sp. WS192  *Ca.* Nitrosocosmicus sp. 48 S62  *Ca.* Nitrosocosmicus sp. 47 S61  *Ca.* Nitrosocosmicus hydrocola G61  *Ca.* Nitrosocosmicus agrestis SS  *Ca.* Nitrosocosmicus articus Kfb  *Ca.* Nitrosocosmicus oleophilus MY3  “*Ca*. Nitrosocosmicus” clade-specific -rpoB primer | TATGGGTTCAAGTACACTGG  TATGGGTTCAAGTACACTGG  CATGGCTTTGAATATTCTGG  CATGGCTTTGAATATTCTGG  CATGGCTTTGAATATTCTGG  CATGGTTTCGAATATTCTGG  CACGGTTTTGAATATTCTGG  CATGGTTTTGAATACTCTGG  CATGGTTTTGAATATTCTGG  CATAATTTCAAATATTCTGG  CACAATTTCAAATATTCTGG  CACAATTTCAAATATTCTGG  CACAATTTCAAATATTCTGG  CATGGTTTCGAATATTCTGG  CATGGTTTCAAATATTCTGG  GCTGGTTTCAAATATTCTGG  GCTGGATTCAAATATTCTGG  CAAGGTTTCAAGTATTCTGG  TCTGGACTGAAATATTCTGG  TCTGGTCTGAAATATTCAGG  AGCGGACTGAAATATTCTGG  AGCGGTTTGAAATATTCTGG  AGCGGTTTGAAATATTCTGG  AATGGATTCAAGTATTCTGG  AACGGATTCAAATATTCTGG  AACGGATTCAAGTATTCTGG  AATGGTTTCAAGTATTCTGG  AATGGATTCAAGTATTCTGG  CACGGCTTCAAATATAGCGG  TACGGATTCAAGTACACCGG  TACGGCTTCAAGTACACGGG  TACGGCTTCAAGTACACGGG  TATGGTTTTAAGCACAGCGG  TATGGATTCAAGCATAGCGG  TACGGATTCAAGCATAGTGG  TACGGATTCAAGCACAGTGG  TATGGATTCAAGCATAGTGG  TATGGATTCAAGCATAGTGG  TATGGATTCAAGCACAGTGG  TATGGTTTTAAGCACAGCGG  TATGGATTCAAGCATAGCGG  TACGGATTCAAGCATAGTGG  TAYGGWTTYAAGCAYAGTGG | TGAGCCTCAACATACTCCC  TGAGCCTCAACATACTCCC  AGAGTCTCAATGTCGCACC  AGAGCCTCAACGTCGCACC  AGAGTCTCAATGTTGCACC  AAAGTCTTAACGTTGCACC  AAAGTCTTAACGTTGCACC  AGAGTCTTAACGTTGCACC  AGAGTCTCAATGTTGCACC  AAAGTCTTAATGTTGCACC  AAAGTCTAAACGTTGCACC  AAAGTCTTAACGTTGCACC  AGAGTCTTAATGTCGCACC  AGAGTCTTAATGTTGCACC  AGAGTCTTAACGTTGCACC  AAAGTTTGAATGTGGCCCC  AAAGTTTGAATGTGGCTCC  TAAGTCTTAACATCGCACC  AAAGCCTCAACGTAGCGCC  AGAGCCTTAACGTAGCACC  AAAGCCTCAATGTGGCCCC  AGAGCCTCAACGTGGCGCC  AGAGCCTCAACGTGGCGCC  TGAGTCTTGATGTGGCACC  TGAGTCTTGATGTCGCACC  TGAGTCTTGATGTGGCACC  TGAGTCTTGATGTTGCACC  TGAGTCTTGATGTAGCACC  TGAGTCTTAACATCGCTCC  TGAGCCTGAACGTCGCTCC  TGAGCCTCAACGTCGCTCC  TGAGCCTCAACGTCGCTCC  TGAGTTTAAATGTGGCTCC  TGAGTTTAAATGTGGCTCC  TGAGTTTAAATGTCGCTCC  TGAGTTTAAATGTGGCACC  TGAGTTTAAATGTCGCTCC  TGAGTTTAAATGTCGCTCC  TGAGTTTAAATGTGGCGCC  TGAGTTTAAATGTGGCTCC  TGAGTTTAAATGTCGCTCC  TGAGTTTAAATGTCGCTCC  TGAGTTTAAATGTSGCWCC |

NS-α-3

NT-α-1

NP-η-1

NP-ε-2

NP-γ-2.2

NP-γ-2.1

NC-α

NS-ζ

# Fig. S9: Sequences of “*Ca.* Nitrosocosmicus” clade-specific *rpoB* gene primers

AOA *rpoB* gene sequences were aligned for primer design. Nucleotide residues of reference sequences that matched to primer sequences are highlighted in dark yellow, and partially matched nucleotide residues are highlighted in dark cyan. The sequence name of “*Ca.* Nitrosocosmicus” clade-specific *rpoB* gene primers is highlighted in yellow

# Supplementary References

1. Bulgarelli D, Rott M, Schlaeppi K, Ver Loren van Themaat E, Ahmadinejad N, Assenza F, Rauf P, Huettel B, Reinhardt R, Schmelzer E, Peplies J, Gloeckner FO, Amann R, Eickhorst T, Schulze-Lefert P. 2012. Revealing structure and assembly cues for *Arabidopsis* root-inhabiting bacterial microbiota. Nature 488:91-95.

2. Peiffer JA, Spor A, Koren O, Jin Z, Tringe SG, Dangl JL, Buckler ES, Ley RE. 2013. Diversity and heritability of the maize rhizosphere microbiome under field conditions. Proc Natl Acad Sci USA 110:6548-6553.

3. Uroz S, Buée M, Murat C, Frey-Klett P, Martin F. 2010. Pyrosequencing reveals a contrasted bacterial diversity between oak rhizosphere and surrounding soil. Environ Microbiol Rep 2:281-288.

4. Edwards J, Johnson C, Santos-Medellín C, Lurie E, Podishetty NK, Bhatnagar S, Eisen JA, Sundaresan V. 2015. Structure, variation, and assembly of the root-associated microbiomes of rice. Proc Natl Acad Sci USA 112:E911-E920.

5. Schreiter S, Ding G-C, Heuer H, Neumann G, Sandmann M, Grosch R, Kropf S, Smalla K. 2014. Effect of the soil type on the microbiome in the rhizosphere of field-grown lettuce. Front Microbiol 5:144.

6. Yeoh YK, Paungfoo-Lonhienne C, Dennis PG, Robinson N, Ragan MA, Schmidt S, Hugenholtz P. 2016. The core root microbiome of sugarcanes cultivated under varying nitrogen fertilizer application. Environ Microbiol 18:1338-1351.

7. Parks DH, Chuvochina M, Chaumeil P-A, Rinke C, Mussig AJ, Hugenholtz P. 2020. A complete domain-to-species taxonomy for Bacteria and Archaea. Nat Biotechnol 38:1079-1086.

8. Tourna M, Stieglmeier M, Spang A, Könneke M, Schintlmeister A, Urich T, Engel M, Schloter M, Wagner M, Richter A, Schleper C. 2011. *Nitrososphaera viennensis*, an ammonia oxidizing archaeon from soil. Proc Natl Acad Sci USA 108:8420-8425.

9. Klindworth A, Pruesse E, Schweer T, Peplies J, Quast C, Horn M, Glöckner FO. 2012. Evaluation of general 16S ribosomal RNA gene PCR primers for classical and next-generation sequencing-based diversity studies. Nucleic Acids Res 41:e1.

10. Osburn MR, Sessions AL, Pepe-Ranney C, Spear JR. 2011. Hydrogen-isotopic variability in fatty acids from Yellowstone National Park hot spring microbial communities. Geochim Cosmochim Acta 75:4830-4845.

11. Kalyaanamoorthy S, Minh BQ, Wong TKF, von Haeseler A, Jermiin LS. 2017. ModelFinder: fast model selection for accurate phylogenetic estimates. Nat Methods 14:587-589.

12. Nguyen L-T, Schmidt HA, von Haeseler A, Minh BQ. 2015. IQ-TREE: a fast and effective stochastic algorithm for estimating maximum-likelihood phylogenies. Mol Biol Evol 32:268-274.

13. DeSantis TZ, Hugenholtz P, Larsen N, Rojas M, Brodie EL, Keller K, Huber T, Dalevi D, Hu P, Andersen GL. 2006. Greengenes, a Chimera-Checked 16S rRNA Gene Database and Workbench Compatible with ARB. Appl Environ Microbiol 72:5069-5072.
